# Untargeted metabolomics analysis of esophageal squamous cell cancer progression

**DOI:** 10.1186/s12967-022-03311-z

**Published:** 2022-03-14

**Authors:** Tao Yang, Ruting Hui, Jessica Nouws, Maor Sauler, Tianyang Zeng, Qingchen Wu

**Affiliations:** 1 Department of Thoracic and Cardiovascular Surgery, The First Affiliated Hospital of Chongqing Medical University, Chongqing, 400016 China; 2 Department of Rehabilitation Medicine, Chengdu First Peoples’ Hospital, Chengdu, 610016 Sichuan China; 3 Department of Pulmonary, Critical Care and Sleep Medicine, School of Medicine of Yale University, New Haven, CT 06510 USA

**Keywords:** Esophageal squamous cell carcinoma, Metabolomics, Glycerophospholipid metabolism, PTDSS1, LPCAT1

## Abstract

90% of esophageal cancer are esophageal squamous cell carcinoma (ESCC) and ESCC has a very poor prognosis and high mortality. Nevertheless, the key metabolic pathways associated with ESCC progression haven’t been revealed yet. Metabolomics has become a new platform for biomarker discovery over recent years. We aim to elucidate dominantly metabolic pathway in all ESCC tumor/node/metastasis (TNM) stages and adjacent cancerous tissues. We collected 60 postoperative esophageal tissues and 15 normal tissues adjacent to the tumor, then performed Liquid Chromatography with tandem mass spectrometry (LC–MS/MS) analyses. The metabolites data was analyzed with metabolites differential and correlational expression heatmap according to stage I vs. con., stage I vs. stage II, stage II vs. stage III, and stage III vs. stage IV respectively. Metabolic pathways were acquired by Kyoto Encyclopedia of Genes and Genomes. (KEGG) pathway database. The metabolic pathway related genes were obtained via Gene Set Enrichment Analysis (GSEA). mRNA expression of ESCC metabolic pathway genes was detected by two public datasets: gene expression data series (GSE)23400 and The Cancer Genome Atlas (TCGA). Receiver operating characteristic curve (ROC) analysis is applied to metabolic pathway genes. 712 metabolites were identified in total. Glycerophospholipid metabolism was significantly distinct in ESCC progression. 16 genes of 77 genes of glycerophospholipid metabolism mRNA expression has differential significance between ESCC and normal controls. Phosphatidylserine synthase 1 (PTDSS1) and Lysophosphatidylcholine Acyltransferase1 (LPCAT1) had a good diagnostic value with Area under the ROC Curve (AUC) > 0.9 using ROC analysis. In this study, we identified glycerophospholipid metabolism was associated with the ESCC tumorigenesis and progression. Glycerophospholipid metabolism could be a potential therapeutic target of ESCC progression.

**Supplementary Information:**

The online version contains supplementary material available at 10.1186/s12967-022-03311-z.

## Background

Esophageal cancer is the sixth leading cause of cancer deaths and one of the poorly understood cancers in the world [1–3]. Approximately, 90% of esophageal cancer is esophageal squamous cell carcinoma (ESCC) [4, 5]. ESCC has a very poor prognosis and high mortality [6], in part because ESCC is usually detected by enhanced thoracic computerized tomography (CT) and gastroscopy at late disease stages.

Metabolomics has become a new platform for biomarker discovery over recent years [7, 8]. Metabolic profiling of Liquid Chromatography with tandem mass spectrometry (LC–MS/MS) can be used to measure multiple metabolic changes simultaneously during pathological processes and identify the dynamic metabolic response of vital intermediary biochemical pathways [9–11]. Metabolomics detection has been applied for a range of cancers, including esophageal cancer [12–14], brain [15], gastric [16], breast [17], bladder [18], lung [19], and thyroid [20]. Zhu et al*.* utilized LC–MS/MS to ESCC patients metabolomics via plasma and found eight metabolites panel can be as potential diagnostic biomarkers and indoleacrylic acid, Lysophosphatidylcholine (LPC) (20:5), and Lysophosphatidylethanolamine (LPE) (20:4) were related to the ESCC progression [12]. Tokunaga et al. used capillary electrophoresis time-of-flight mass spectrometry to esophageal cancer and found tricarboxylic acid cycle activity downregulation in pT3-4 compared to pT1-2 [13]. Chen et al. found tryptophan, formylkynurenine, kynurenine and indoleamine 2,3-dioxygenase 1 as potential therapeutic targets for ESCC through LC–MS/MS [14]. However, there are no studies exploring all tumor/node/metastasis (TNM) stages metabolic features via ESCC cancerous tissues, thus the key metabolic pathways in ESCC progression haven’t been revealed yet.

This study aimed to determine distinguished metabolites and metabolic pathways for ESCC progression. We use the ultra-high-performance LC–MS/MS analysis of all ESCC TNM stages and normal tissues to the tumor to elucidate the aberrant metabolic pathways and to provide insights into ESCC progression.

## Methods

### Patients and clinical characteristics

We collected a total of 75 esophageal tissues, including 15 samples of esophageal squamous cell cancer (ESCC) TNM stage I, 15 samples of ESCC TNM stage II, 15 samples of ESCC TNM stage III, 15 samples of ESCC TNM stage IV, and 15 samples of normal tissues adjacent to the tumor in this study. All esophageal samples were from the Frist Affiliated Hospital of Chongqing Medical University, China, from January 2010 to December 2019. The characteristics of these patients were shown in Table 1. The patients were diagnosed with ESCC by preoperative gastroscopy and subsequently recruited for the study. The esophageal tissues were acquired during the gastroscopic biopsy or surgery and were applied for pathologic biopsy. The tumor segments enrolled meet the following criteria: viable tumor nuclei > 80%, total cellularity > 50%, and necrosis < 20% [21, 22]. Normal tissues adjacent to the tumor were acquired by other 15 ESCC patients, all the patients had no illness of esophagitis, acid reflux, or gastritis, and no patients received the chemoradiation therapy before the surgery. All included patients signed informed consent before they participated in the study. The study was implemented in terms of the Declaration of Helsinki, and the study was approved by the Ethics Committee of the First Affiliated Hospital of Chongqing Medical University. All tissue samples were immediately stored at frozen.

**Table 1:** Clinicopathological characteristics of esophageal squamous cell carcinoma patients

Characteristics	Esophageal squamous cell carcinoma TNM Stage				
	TNM Stage I (N = 15)	TNM Stage II (N = 15)	TNM Stage III (N = 15)	TNM Stage IV (N = 15)	Adjacent cancerous tissues (N = 15)
Age					
< 65—no. (%)	6 (40.0)	5 (33.3)	9 (60.0)	7 (46.7)	11 (73.3)
≥ 65—no. (%)	9 (60.0)	10 (66.7)	6 (40.0)	8 (53.3)	4 (26.7)
Genders					
Female—no. (%)	3 (20.0)	4 (26.7)	1 (6.7)	2 (13.3)	5 (33.3)
Male—no. (%)	12 (80.0)	11 (73.3)	14 (93.3)	13 (86.7)	10 (66.7)
Body-mass index					
Median (IQR)	20.8 (19.3–22.5)	20.3 (18.2–22.4)	19.2 (18.2–20.6)	18.6(17.6–19.2)	21.3 (19.9–23.2)
Tumor location^a^					
Lt—no. (%)	3 (20.0)	5 (33.3)	9 (60.0)	6 (40.0)	
Mt—no. (%)	10 (66.7)	7 (46.7)	5 (33.3)	7 (46.7)	
Ut—no. (%)	2 (13.3)	3 (20.0)	1 (6.7)	2 (13.3)	

*Lt* lower thoracic esophagus, *Mt* middle thoracic esophagus, *Ut* upper thoracic esophagus

^a^Tumor location was classified according to the Union for International Cancer Control Tumor, Node, Metastasis cancer staging system (ninth edition)

### Sample preparation and extraction

We performed sample preparation and extraction as previously described [23]. We weighed 25 mg of the sample in an EP tube and added 500 μL of extraction solution (acetonitrile: methanol: water = 2:2:1, a standard internal mixture with isotope labeling). After vortexing for 30 s, we homogenized the samples at 35 Hz for 4 min and then sonicated on ice for 5 min. We repeated the homogenization and sonication cycle three times. The samples were then incubated at − 40 °C for 1 h and centrifuged at 12,000 rpm at 4 °C for 15 min. The supernatant was transferred to a fresh tube for analysis. We prepared quality control (QC) samples by mixing aliquots of the supernatant from all samples.

### UHPLC-QE-MS analysis

LC–MS/MS analyses were performed using a UHPLC system (Vanquish, Thermo Fisher Scientific) and a UPLC BEH Amide column (2.1 mm × 100 mm, 1.7 μm) combined with a Q Exactive HFX mass spectrometer (Orbitrap MS, Thermo). The mobile phase consists of 25 mmol/L ammonium acetate and 25 ammonium hydroxide aqueous solution (pH = 9.75) (A) and acetonitrile (B). The analysis was performed with an elution gradient as follows: 0–0.5 min, 95% B; m/z. 0.5–7.0 min, 95–65% B; 7.0–8.0 min, 65–40% B; 8.0–9.0 min, 40% B; 9.0–9.1 min, 40–95% B; 9.1–12.0 min, 95% B. The column temperature was 25 °C. The temperature of the auto-sampler was 4 °C, and the injection volume was 3 μL. The QE HFX mass spectrometer was used because it can acquire MS/MS spectra in information dependent acquisition (IDA) mode under the control of acquisition software (Xcalibur, Thermo). In this mode, the acquisition software continuously evaluates the full scan MS spectrum. The ESI source conditions were set as follows: sheath gas flow rate was 50 Arb, the auxiliary gas flow rate was 10 Arb, the capillary temperature was 320 °C, full MS resolution was 60,000, MS/MS resolution was 7500, collision energy was 10/30/in NCE It is 60 in the mode, and the spraying voltage was 3.5 kV (positive) or − 3.2 kV (negative).

### Qualitative and quantitative analysis of metabolites

We used proteowizard (http://proteowizard.sourceforge.net/) [24] to convert the original data into mzXML format and used an internal program for processing, which was developed using R and based on package XCMS (version 3.7.1) for peak detection, extraction, alignment, and integration. Then the internal mass-spectrometry 2 (MS2) database (BiotreeDB) was applied to metabolite annotation. The cutoff value of the annotation was set to 0.3.

### Differentially expressed metabolites selection

In this study, principal component analysis (PCA) and orthogonal projection to least squares discriminant analysis (OPLS-DA) were utilized to simplify the metabolomic data. Therefore, after mean-centering and scaling, the UHPLC data were set to default unit variance, multivariate statistical analysis [25–27] was conducted via SIMCA-P version 16.0 software package (Umetrics Umeå, Sweden). First, we performed unsupervised PCA to observe the inner clusters and find apparent outliers. Then, supervised OPLS-DA was used to distinguish the ESCC samples from the adjacent normal controls visually. The OPLS-DA model eliminates variability unrelated to class separation. The quality and reliability of the model were evaluated by the parameters R^2^X, R^2^Y and Q^2^. R^2^X and R^2^Y represent the explained data change and R^2^X indicates the goodness of the fit, R^2^Y indicates goodness of prediction, while Q^2^ is a sevenfold cross-validation parameter and estimates the predictive ability, with aggregate (cum) values of R2X, R2Y and Q2Y equating to ~ 1 showing a valid model. The cumulative value of the total explained value R2X, R2Y and the predictable change of Q2 suggest that the modeling is correct. To prevent over-fitting, a permutation test (n = 200) in the SIMCA-P software package was applied to the OPLS-DA model [28, 29]. Based on the variable importance of the prediction (VIP) threshold from the OPLS-DA model, the metabolites responsible for ESCC differential from adjacent normal esophageal metabolites can be identified. The Mev (MultiExperiment Viewer) 4.8 software was used to execute one-way ANOVA with standard Bonferroni correction to correct the resultant *p* values for each metabolite in all cross-comparisons. By VIP > 1.0 and adjust. *p* value (*p*FDR) < 0.05 (confidence level), all ESCC TNM stages was screened for significantly different metabolites when compared with the adjacent normal esophagus[30]. The pheatmap package and corrplot package in R 3.6.3 were used to draw heatmap and correlation matrix among ESCC TNM stage I vs. con., stage I vs. stage II, stage II vs. stage III, and stage III vs. stage IV [31, 32].

### Metabolic pathway enrichment and pathway related-genes analysis

According to the Kyoto Encyclopedia of Genes and Genomes (KEGG) metabolites compounds database [33], we annotated verified metabolites of ESCC TNM stage I vs. con., stage I vs. stage II, stage II vs. stage III, stage III vs. stage IV and then matched the annotated metabolites with the KEGG pathway database. We fed the most significantly regulated metabolite pathway into gene set enrichment analysis (GSEA) [34]. We next analyzed the metabolic pathway genes mRNA expression between ESCC and normal esophageal tissues in public datasets: gene expression data series (GSE)23400 and The Cancer Genome Atlas (TCGA) [35, 36]. Receiver operating characteristic (ROC) curves applied to assess the ESCC progression predictive value by GSE23400 dataset [37].

## Results

### Patients’ characteristics

From January 2010 through December 2019, 75 esophageal tissues were collected including esophageal squamous cell carcinoma (ESCC) and normal tissues adjacent to the tumor. Compared with either ESCC tumor/node/metastasis (TNM) stages and normal controls, more than 70% ESCC patients were male, almost 80% ESCC located at middle and lower esophagus. 65% ESCC Stage II patients were older than 65, and ESCC Stage IV patients had lowest BMI among all groups (IQR, 17.6–19.2). Characteristics of patients see Table 1.

### Metabolic profiles of ESCC and normal esophageal tissues

To assess all ESCC TNM stages and adjacent cancerous tissues metabolic profiles, we measured esophageal tissues via LC–MS/MS. Finally, 712 metabolites over 75 classes were identified, including 145 Glycerophospholipids, 124 Carboxylic acids and (its) derivatives, and 84 Fatty Acyls et al. (Additional file 1 Identified Metabolites: Table S1). Phosphatidylethanolamine (PE) and phosphatidylcholine (PC) were mostly Glycerophospholipids species. The data that support the findings of this study have been deposited into MetaboLights of EMBL-EBI with MTBLS3579 [38].

### Multivariate statistics analysis

**Fig. 1:**
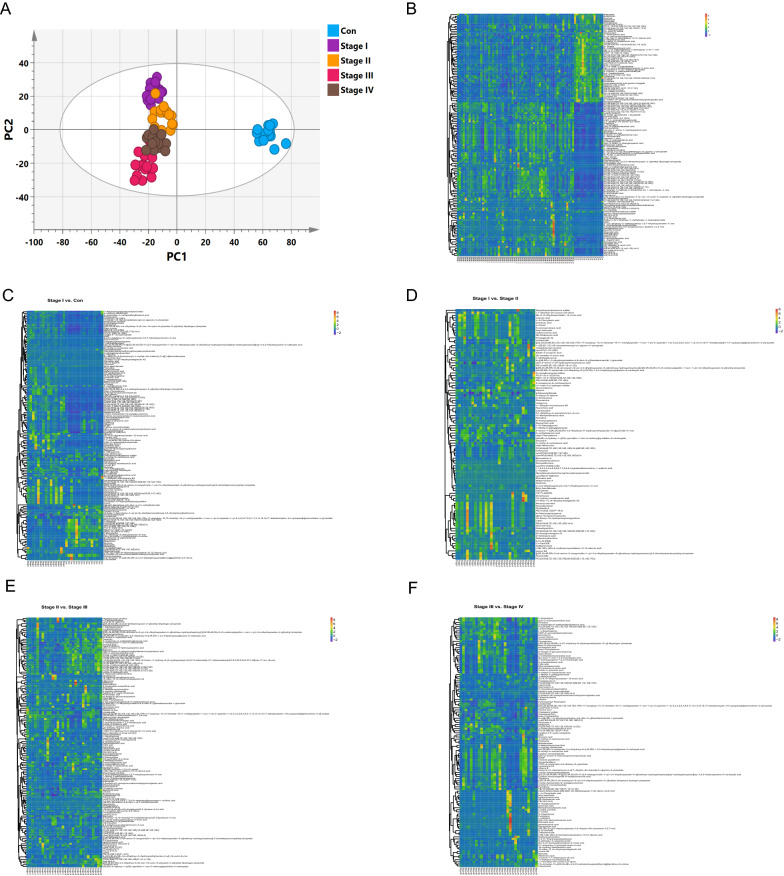
Metabolite’s profiling of all TNM stages of ESCC and normal tissues adjacent to the tumor. **A** OPLS-DA score plot. Heatmap of metabolites expression. **B** All TNM stages vs. con., **C** stage I vs. con., **D** stage I vs. stage II, **E** stage II vs. stage III, **F** stage III vs. stage IV

Principle component analysis (PCA), and orthogonal partial least squares discriminant analysis (OPLS-DA) as data mining methods are used to build multivariate models to discriminate metabolomic profiling among ESCC TNM stages and adjacent cancerous tissues [39]. PCA score plots showed a clear trend of group clusters between the ESCC patients and normal controls (Additional file 1 PCA: Fig. S1). Additionally, to exclude variables with smaller correlations, a supervised OPLS-DA classification model using one PLS component and one orthogonal component was established. The OPLS-DA score plots obtained even clearer class discrimination (Fig. 1A). Goodness of fit (R2 X and R2Y) of ESCC TNM stages versus controls were 0.479 and 0.912, and Q2 of OPLS-DA was 0.815. These results indicated 712 metabolites were well explained by OPLS-DA models. To validate the OPLS-DA models, random permutation tests with 200 permutations were performed (Additional file 1: Validation plots. Fig. S2). Decrease of Q^2^ and R^2^ was observed along with the decrease of *X*-axis value, suggesting the model did not overfit. The PCA and OPLS-DA plots showed good discrimination between all ESCC TNM stages and normal controls.

### Differential metabolites screening

To differentiate specific metabolites among all ESCC TNM stages vs. adjacent normal controls, OPLS-DA results were used to screen all metabolites with significant differences. The Variable Importance in Projection (VIP) obtained from OPLS-DA reflects both the loading weights for each sample and the metabolite of the response explained by this sample and can be used for metabolite selection. In this study, the metabolites concentration VIP value (VIP > 1) combined with the metabolite’s concentration adjust *p*-value (*p*FDR < 0.05) was used to screen the crucial metabolites. As a result, 145 metabolites had significant differences among all ESCC TNM stages vs. Con.; the ESCC TNM stage I vs. Con. group had 151 metabolites with significant differences; the ESCC TNM stage I vs. stage II group had 100 metabolites with significant differences; the ESCC TNM stage II vs. stage III group had 144 metabolites with significant differences; and the ESCC TNM stage III vs. stage IV group had 120 metabolites with significant differences. Heatmaps were plotted using z-score among all ESCC TNM groups and normal control group.

### Metabolite’s correlation analysis in ESCC progression

**Fig. 2:**
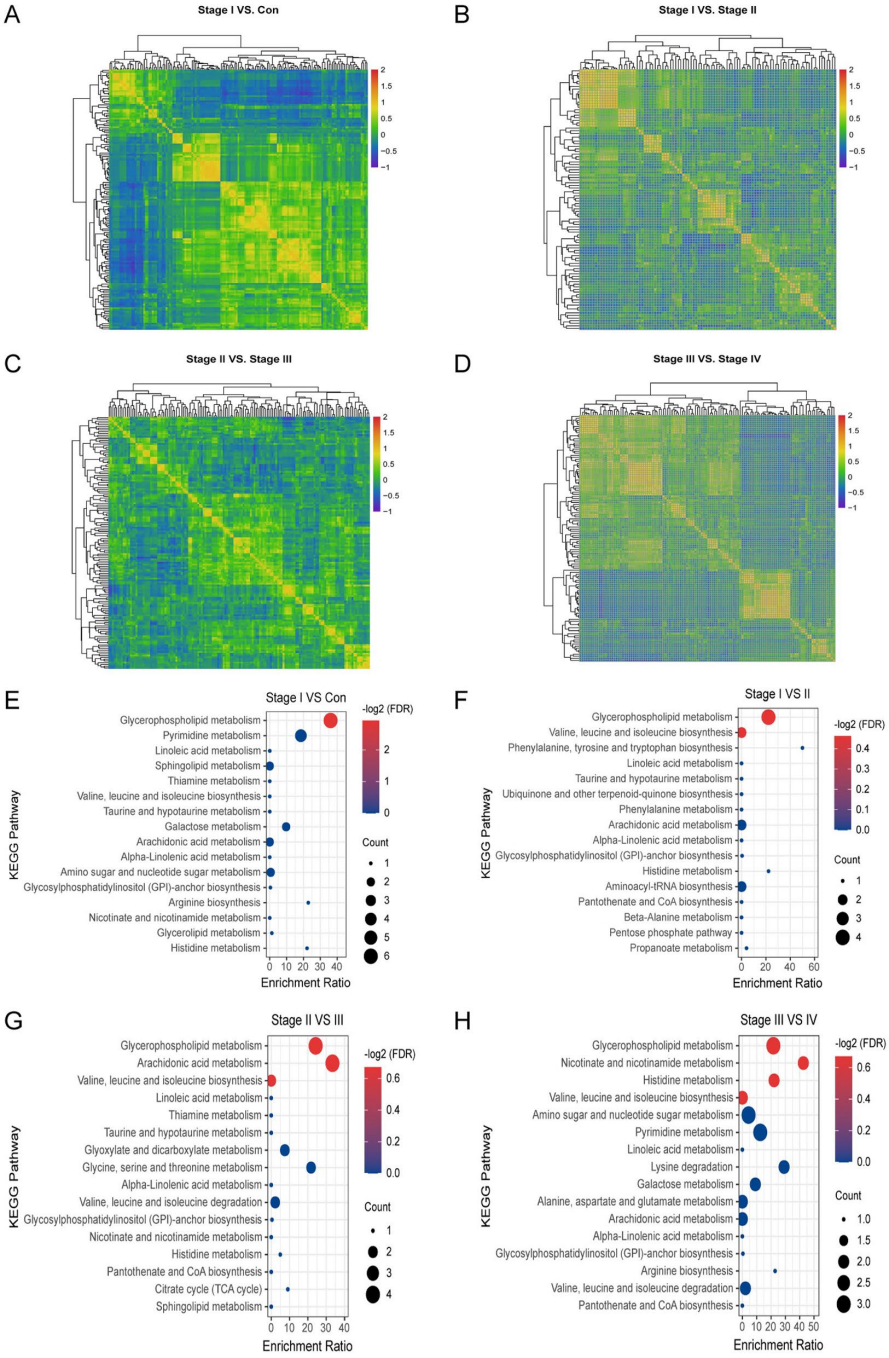
Metabolite’s expression correlational heatmap and KEGG pathway analysis of ESCC aggressiveness. Pearson correlation coefficient analysis of significantly differential expression metabolites of all ESCC TNM stages vs. adjacent normal controls. **A** stage I vs. con., **B** stage I vs. stage II. **C** Stage II vs. stage III, **D** stage III vs. stage IV. KEGG pathway of all ESCC TNM stages vs. adjacent normal controls. **E** Stage I vs. con., **F** stage I vs. stage II. **G** Stage II vs. stage III, **H** stage III vs. stage IV

Pearson correlation coefficient analysis was applied for metabolite-metabolite correlation analysis via z-score in all ESCC TNM Stages vs. Con. This analysis identified metabolites that associated with each other in esophageal carcinoma and normal controls. Specifically, we compared metabolite correlations between each pair of samples (stage I vs. con., stage I vs. stage II, stage II vs. stage III, and stage III vs. stage IV), and the metabolite-metabolite correlations of these three sample combinations showed unique profiles. Metabolites with correlation coefficients *p* < 0.1 was identified as significantly correlation (Fig. 2A–D).

### KEGG pathway related to ESCC Progression

To validate ESCC progression related metabolic pathway, significantly differential metabolites in all ESCC TNM stage vs. Con were retrieved from the KEGG compound database. The results of the KEGG pathway enrichment analysis were displayed 16 pathways in either ESCC TNM stages and adjacent normal control via adjust *p*-value (*p*FDR < 0.05), metabolites count, and enrichment ratio. Among all ESCC TNM stage vs. Con. groups, the Glycerophospholipid metabolism pathway was the most significantly distinct metabolic pathway (Fig. 2E–H).

### Glycerophospholipid metabolism genes analysis

**Fig. 3:**
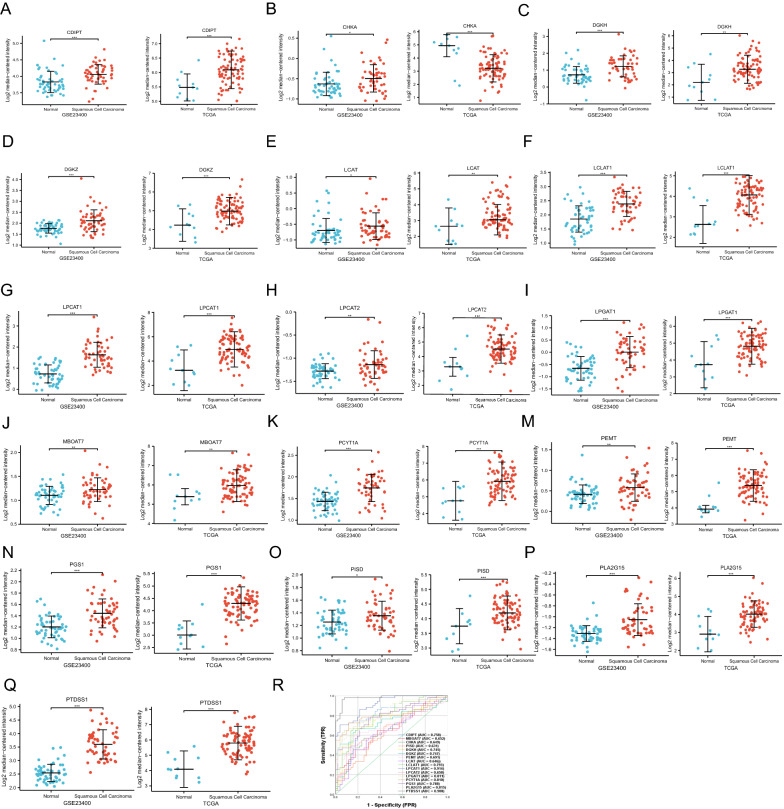
mRNA expression of glycerophospholipid metabolism genes and ROC analysis. 16 genes of 77 Glycerophospholipid metabolism GSEA genes showed differential significance in GSE23400 and TCGA dataset. **A** CDIPT, **B** CHKA, **C** DGKH, **D** DGKZ, **E** LCAT, **F** LCLAT1, **G** LPCAT1, **H** LPCAT2, **I** LPGAT1, **J** MBOAT7, **K** PCYT1A, **M** PEMT, **N** PGS1, **O** PISD, **P** PLA2G15, **Q** PTDSS1. 16 genes of glycerophospholipid metabolism ROC analysis. **R** ROC curves

We obtained the gene set of Glycerophospholipid metabolism genes from the GSEA. A total of 77 genes were retrieved by Glycerophospholipid metabolism (Additional file 1 mRNA Expressions of Glycerophospholipid Metabolism Genes: Table S2). Moreover, we analyzed the 77 genes mRNA expression in normal esophageal tissues and all stages of ESCC tissues in GSE23400 and TCGA dataset and found 16 genes had significantly differential expression (Fig. 3A–Q). Then 16 significantly differential genes of Glycerophospholipid metabolism were performed ROC test from GSE23400. Phosphatidylserine Synthase 1 (PTDSS1) (AUC = 0.980) and Lysophosphatidylcholine Acyltransferase 1 (LPCAT1) (AUC = 0.914) showed a good prediction of ESCC in Fig. 3R.

## Discussion

For metabolic pathways of esophageal squamous cell cancer (ESCC) were still unclear, we embarked on a metabolomics study. In this study, we found 712 metabolites in all tumor/node/metastasis (TNM) ESCC and adjacent normal tissues, in which were 145 glycerophospholipid. Moreover, we found glycerophospholipid metabolism was dominant in all TNM ESCC stages via Kyoto Encyclopedia of Genes and Genomes (KEGG) analysis. Furthermore, glycerophospholipid metabolism was associated with 77 genes, in which16 genes were linked to ESCC. In addition, we generated a receiver operating characteristic curve (ROC) curve for each 16 significantly differential mRNA expression genes in ESCC and reported the Area under the Curve (AUC) for each gene. Phosphatidylserine Synthase 1 (PTDSS1) and Lysophosphatidylcholine Acyltransferase 1 (LPCAT1) had a good diagnostic value with AUC > 0.9. These findings suggested glycerophospholipid metabolism was related to ESCC progression.

In recent years, metabolomic based approaches have been recognized as an emerging tool to discover products of cellular biochemical reactions that fuel cell proliferation in a variety of malignancies. Several studies [40–42] have found distinct differences in the metabolic profile of patients with cancers and related disorders. Pandey et al. utilized Nuclear Magnetic Resonance (NMR) metabolomes to distinguish brain tumors in vitro and vivo and identified Cysteine metabolism as a crucial marker in brain cancer aggressiveness [40]. Jing et al*.* employed liquid chromatography–tandem mass spectrometry (LC–MS/MS) to detect 84 gastric cancer patients and 82 gastric ulcer patients’ plasma samples, and found five differential amino acids, glutamine, ornithine, histidine, arginine, and tryptophan, were identified for discerning between gastric cancer and gastric ulcer [41]. Barberini et al*.* examined pre-treatment plasma samples from 66 adult patients with any lymphoma subtype and 96 frequency-matched population controls and found fatty acids were mostly represented in multiple myeloma and Hodgkin lymphoma patients [42]. Here, we employed LC–MS/MS to detect 75 samples of all TNM stages ESCC and normal tissues adjacent to the tumor metabolomes and identified 712 metabolites and the dominant metabolites were 145 glycerophospholipid. We thus brought insight into how metabolites were involved in ESCC, and these findings contributed new insights for researchers to understand the role of metabolites in ESCC.

**Fig. 4:**
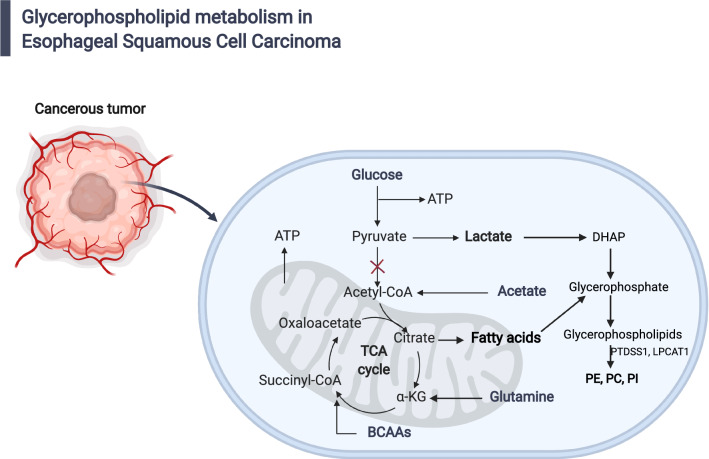
Diagram of glycerophospholipid metabolism in ESCC. Glycerophospholipid metabolic pathway for the biosynthesis of glycerophosphate from lactate and glutamine under tumor cells starvation

Glycerophospholipid metabolism is currently understood as most relevant to cancer development and progression [43–46]. Major glycerophospholipids (GPLs) in the cell include phosphatidylserine (PS), Phosphatidylethanolamine (PE), phosphatidylcholine (PC), phosphatidylinositol (PI), phosphatidic acid (PA), phosphatidylglycerol (PG), and cardiolipin (CL) [47]. Here, PC and PE were identified as the two most abundant GPLs in all ESCC TNM stages and normal control tissues. PE was a critical precursor for PC. Tsigelny et al*.* used metabolomics to study early and late stages of bladder cancer, and they found that glycerophospholipid metabolism was related to late-stage bladder cancer [44]. Ridgway reported phosphatidylcholine and choline metabolites involved in cancer cells signaling or growth pathways and contribute to both proliferative growth and programmed cell death [45]. Uchiyama et al*.* elucidated PC species played an important role in the mechanism of cancer invasion using imaging mass spectrometry [46]. In this study, LC–MS/MS identified glycerophospholipid metabolism was a cofactor that related to ESCC oncogenesis and progression (Fig. 4).

Metabolites of glycerophospholipid metabolism, phosphatidylethanolamine (PE) and phosphatidylcholine (PC), were consistent from stage I to stage IV of ESCC, thus related to ESCC progression. PTDSS1 encodes phosphatidylserine synthase 1 to catalyze a base-exchange reaction in which the polar head group of PE or PC is replaced by l-serine. Zhu et al*.* and Chen et al*.* found PE and PC were associated with ESCC progression and were potential therapeutic target [12, 14]. You-Tyun et al*.* found that phosphatidylserine synthase 1 (PTDSS1) was an oncogene and a potential therapeutic target for lung adenocarcinoma [48]. Lysophosphatidylcholine Acyltransferase1 (LPCAT1) encodes Lysophosphatidylcholine acyltransferase 1 to catalyze the conversion of lysophosphatidylcholine (1-acyl-sn-glycero-3-phosphocholine) into PC. Several studies have shown that in many solid tumors chemoresistance, tumor aggressiveness and worsened survival correlated with LPCAT1 [49–51]. Here, we found lysophosphatidylcholine was abundant in all ESCC TNM stages vs. Con groups. Taken together, glycerophospholipid metabolism was potential diagnosis, severity assessment and therapeutic options of ESCC progression.

However, we also realized that this study has a few limitations. The major weakness is this study only contains Asian population. Thus, the predictive values of the glycerophospholipid metabolism for ESCC should also be replicated in other populations and ethnic groups. Additionally, metabolites changes are informative but do not always reflect protein concentration or function. Collectively, the most advantage of this study is that we investigate metabolome among all TNM stages ESCC versus adjacent normal esophagus.

## Conclusions

Taken together, glycerophospholipid metabolism promotes ESCC progression, and could be a potential therapeutic target for ESCC progression.

## Supplementary Information

**Additional file 1: Figure S1.** Principal component analysis (PCA) of all ESCC TNM Stages and normal esophageal tissues. **Figure S2.** Validation plots of OPLS-DA models using 200 premutation tests. **Table S1.** 712 identified metabolites of all ESCC TNM Stages and normal controls tissues identified by LS-MS/MS. **Table S2.** 77 genes mRNA expression of Glycerophospholipid metabolism among all ESCC TNM stages and adjacent normal control tissues.

## Data Availability

The data that support the findings of this study have been deposited into MetaboLights of EMBL-EBI with MTBLS3579.

## Abbreviations

ESCC: Esophageal squamous cell carcinoma
LC–MS-MS: Liquid chromatography with tandem mass spectrometry
QC: Quality control
IDA: Information-related acquisition
PCA: Principal component analysis
OPLS-DA: Orthogonal projection to least squares discriminant analysis
VIP: Variable importance of the prediction
ROC: Receiver operating characteristic
GPLs: Glycerophospholipids
PS: Phosphatidylserine
PE: Phosphatidylethanolamine
PC: Phosphatidylcholine
PI: Phosphatidylinositol
PA: Phosphatidic acid
PG: Phosphatidylglycerol
CL: Cardiolipin
PTDSS1: Phosphatidylserine synthase 1
LPCAT1: Lysophosphatidylcholine acyltransferase1
