# Supplementary material for: Untargeted metabolomics analysis of esophageal squamous cell cancer progression
Source: J Transl Med. 2022 Mar 14;20:127. doi: 10.1186/s12967-022-03311-z (PMC8919643; doi:10.1186/s12967-022-03311-z)
Supplement: Supplementary file 1 — Additional file 1: Figure S1. Principal component analysis (PCA) of all ESCC TNM Stages and normal esophageal tissues. Figure S2. Validation plots of OPLS-DA models using 200 premutation tests. Table S1. 712 identified metabolites of all ESCC TNM Stages and normal controls tissues identified by LS-MS/MS. Table S2. 77 genes mRNA expression of Glycerophospholipid metabolism among all ESCC TNM stages and adjacent normal control tissues. [file 12967_2022_3311_MOESM1_ESM.docx]

**Supplementary materials**

**Figure S1** Principal component analysis (PCA) of all ESCC TNM Stages and normal esophageal tissues.


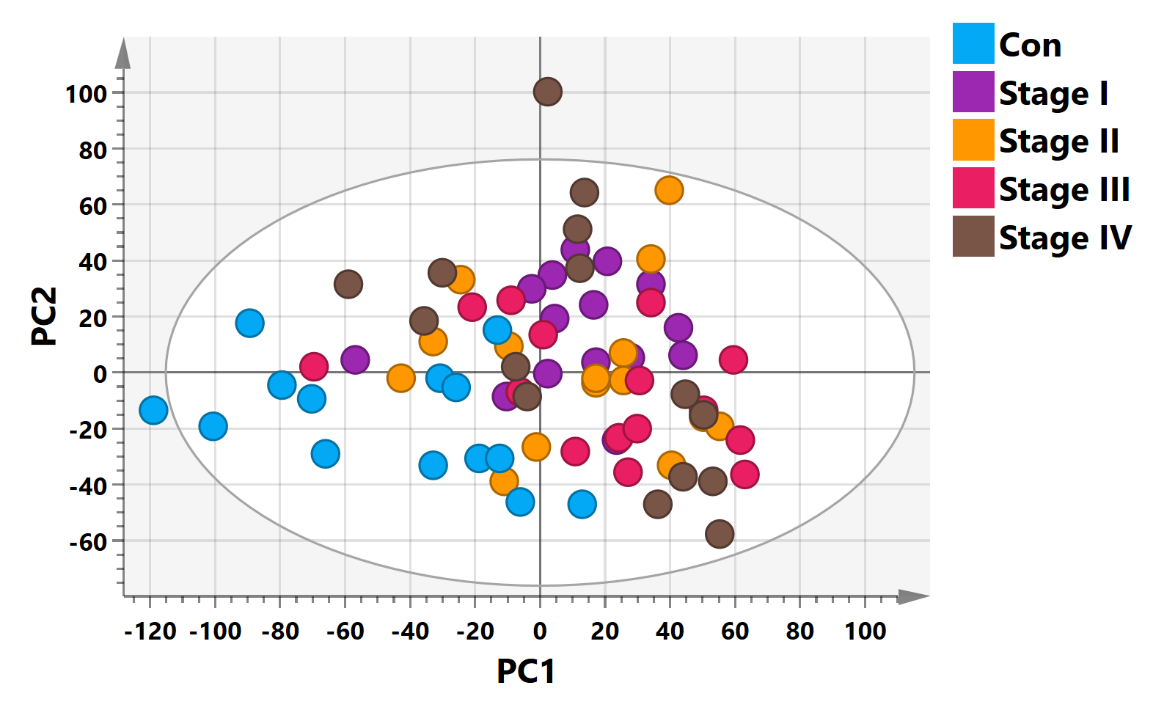


**Figure S2** Validation plots of OPLS-DA models using 200 premutation tests.

**
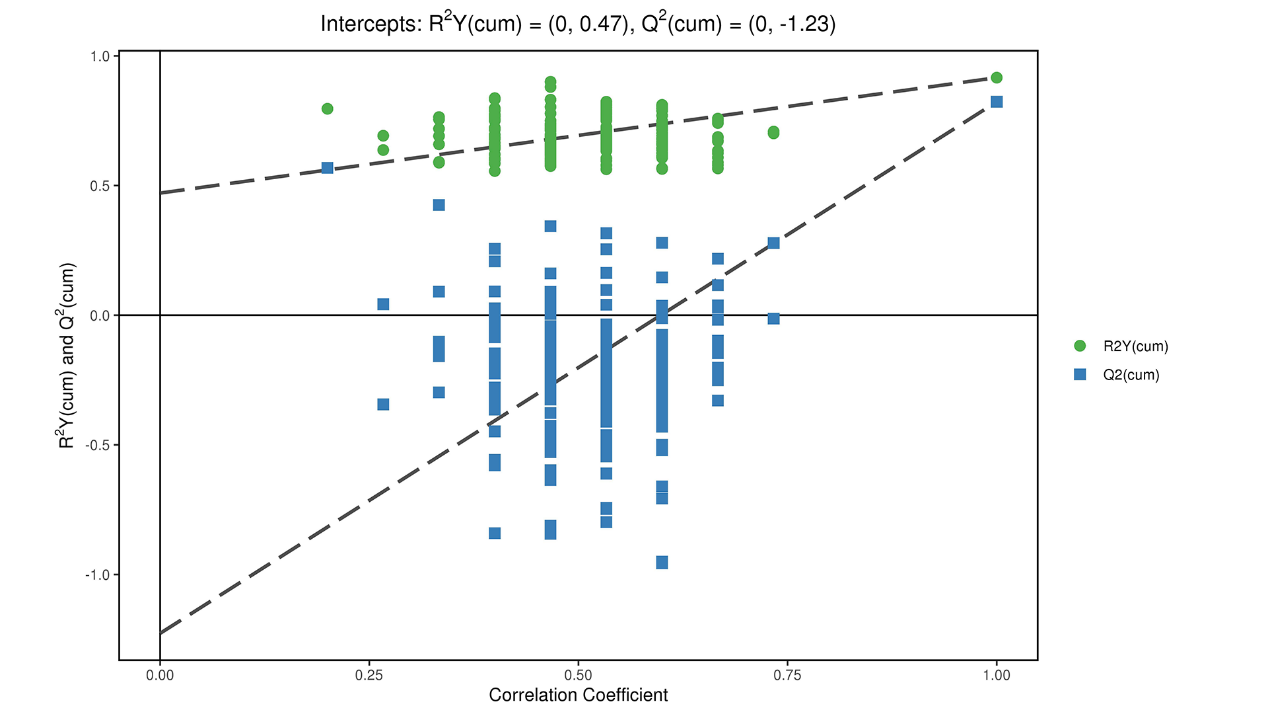
**

**Table S1** 712 identified metabolites of all ESCC TNM Stages and normal controls tissues identified by LS-MS/MS.

| **No.** | **Metabolite** | **MS2 Score** | **rt** | **Mz** | **Super.Class** | **Type** |
| --- | --- | --- | --- | --- | --- | --- |
| 1 | 1-Pyrroline | 0.999994153846154 | 285.65 | 71.06871704 | Organoheterocyclic compounds | POS |
| 2 | Choline | 0.999920307692308 | 374.688 | 104.1068696 | Organic nitrogen compounds | POS |
| 3 | L-Alloisoleucine | 0.999758769230769 | 291.966 | 132.1014125 | Organic acids and derivatives | POS |
| 4 | L-Phenylalanine | 0.999635615384615 | 274.925 | 166.0856433 | Organic acids and derivatives | POS |
| 5 | 2-(trimethylazaniumyl)acetate | 0.999513230769231 | 289.1975 | 118.0859407 | Organic acids and derivatives | POS |
| 6 | Fomepizole | 0.999513076923077 | 705.77 | 82.94502434 | Organoheterocyclic compounds | POS |
| 7 | Methylguanidine | 0.999489153846154 | 706.71 | 74.09666011 | Organic nitrogen compounds | POS |
| 8 | L-Carnitine | 0.999373769230769 | 371.97 | 162.1114741 | Organic nitrogen compounds | POS |
| 9 | 3beta,6beta-Dihydroxynortropane | 0.999370615384615 | 267.857 | 144.1126381 | Alkaloids and derivatives | POS |
| 10 | Cohibin B | 0.999344846153846 | 215.0195 | 577.5167569 | Lipids and lipid-like molecules | POS |
| 11 | 1-methylpyridin-1-ium-3-carboxamide | 0.999326153846154 | 317.9305 | 137.0784234 | Organoheterocyclic compounds | POS |
| 12 | 3-Methyladenine | 0.999297076923077 | 252.373 | 150.0768413 | Organoheterocyclic compounds | POS |
| 13 | Methyl 2-(10-heptadecenyl)-6-hydroxybenzoate | 0.999017769230769 | 55.8039 | 390.2580151 | Benzenoids | POS |
| 14 | 4-Hydroxy-2-butenoic acid gamma-lactone | 0.998933153846154 | 350.3015 | 84.91136897 | Organoheterocyclic compounds | POS |
| 15 | D-Fructosazine | 0.998898538461538 | 346.863 | 321.12908 | Organoheterocyclic compounds | POS |
| 16 | 8,12-Epoxy-4(15),7,11-eudesmatrien-1-one | 0.998728307692308 | 40.36605 | 231.1423213 | Lipids and lipid-like molecules | POS |
| 17 | PE-NMe(16:0/16:0) | 0.998689384615384 | 37.93515 | 706.536446 | Lipids and lipid-like molecules | POS |
| 18 | (2S)-2-amino-3-(1H-indol-3-yl)propanoic acid | 0.998613769230769 | 277.546 | 205.0963532 | Organoheterocyclic compounds | POS |
| 19 | 33-Deoxy-33-hydroperoxyfurohyperforin | 0.998458076923077 | 56.5574 | 569.3882225 | Lipids and lipid-like molecules | POS |
| 20 | pyrrolidine-2-carboxylic acid | 0.998444615384615 | 318.848 | 115.0864736 | Organic acids and derivatives | POS |
| 21 | 2-amino-3-hydroxypropanoic acid | 0.998363230769231 | 381.927 | 105.9954951 | Organic acids and derivatives | POS |
| 22 | 2-Amino-3,4-dimethylimidazo[4,5-f]quinoline | 0.998318307692308 | 41.4947 | 213.1125124 | Organoheterocyclic compounds | POS |
| 23 | Vaccenyl carnitine | 0.998290692307692 | 195.493 | 425.8117231 | Lipids and lipid-like molecules | POS |
| 24 | 2,5-Dichloro-carboxymethylenebut-2-en-4-olide | 0.998247615384615 | 381.944 | 208.9376888 | Organoheterocyclic compounds | POS |
| 25 | Cohibin C | 0.998177923076923 | 161.774 | 577.5168474 | Lipids and lipid-like molecules | POS |
| 26 | Beta-Carboline | 0.998144538461538 | 54.2195 | 169.1328381 | Organoheterocyclic compounds | POS |
| 27 | Cytidine monophosphate | 0.998078076923077 | 428.746 | 323.1003067 | Nucleosides, nucleotides, and analogues | POS |
| 28 | Dicyclohexyl disulfide | 0.998064692307692 | 188.5585 | 230.9510066 | Organosulfur compounds | POS |
| 29 | trans-Hexadec-2-enoyl carnitine | 0.997908692307692 | 195.509 | 398.3253576 | Lipids and lipid-like molecules | POS |
| 30 | Cyclopassifloside II | 0.997777230769231 | 30.0015 | 682.5376099 | Lipids and lipid-like molecules | POS |
| 31 | 6-amino-1H-pyrimidin-2-one | 0.997329153846154 | 267.903 | 112.0503064 | Organoheterocyclic compounds | POS |
| 32 | L-Palmitoylcarnitine | 0.996905 | 194.6635 | 400.3407418 | Lipids and lipid-like molecules | POS |
| 33 | 9-[(2R,3R,4S,5R)-3,4-dihydroxy-5-(hydroxymethyl)oxolan-2-yl]-1H-purin-6-one | 0.996351 | 217.609 | 269.1246152 | Nucleosides, nucleotides, and analogues | POS |
| 34 | 2-Piperidinone | 0.996242153846154 | 52.4954 | 100.0756187 | Organoheterocyclic compounds | POS |
| 35 | Cohibin A | 0.996210384615385 | 163.9315 | 549.4869278 | Lipids and lipid-like molecules | POS |
| 36 | Citrulline | 0.996073153846154 | 364.901 | 176.0397274 | Organic acids and derivatives | POS |
| 37 | Avocadynofuran | 0.995808230769231 | 34.4245 | 247.2043992 | Organoheterocyclic compounds | POS |
| 38 | Pyrrolidine | 0.995628384615385 | 202.1975 | 73.03987136 | Organoheterocyclic compounds | POS |
| 39 | Montecristin | 0.995472538461538 | 32.6582 | 575.5027812 | Lipids and lipid-like molecules | POS |
| 40 | (2E,8Z)-Decadiene-4,6-diyn-1-yl 3-methylbutanoate | 0.995440923076923 | 159.539 | 231.2499612 | Lipids and lipid-like molecules | POS |
| 41 | 3-propanoyloxy-4-(trimethylazaniumyl)butanoate | 0.995269615384615 | 307.251 | 218.107057 | Lipids and lipid-like molecules | POS |
| 42 | SM(d16:1/24:1(15Z)) | 0.994993615384615 | 201.723 | 785.5602049 | Organic nitrogen compounds | POS |
| 43 | L-Arginine | 0.994979769230769 | 541.066 | 174.1229935 | Organic acids and derivatives | POS |
| 44 | PC(24:1(15Z)/18:3(6Z,9Z,12Z)) | 0.994974153846154 | 143.484 | 866.6559202 | Lipids and lipid-like molecules | POS |
| 45 | 2-aminobutanedioic acid | 0.994782461538462 | 414.968 | 134.0443348 | Organic acids and derivatives | POS |
| 46 | Ephedrine | 0.994698615384615 | 202.3955 | 166.1220523 | Benzenoids | POS |
| 47 | PC(20:5(5Z,8Z,11Z,14Z,17Z)/20:3(5Z,8Z,11Z)) | 0.994498461538461 | 144.443 | 830.5658955 | Lipids and lipid-like molecules | POS |
| 48 | N-a-Acetyl-L-arginine | 0.994424846153846 | 384.089 | 217.1037479 | Organic acids and derivatives | POS |
| 49 | 4-Hydroxystachydrine | 0.994151538461538 | 317.05 | 159.112129 | Organic acids and derivatives | POS |
| 50 | (2S)-2-aminopentanoic acid | 0.994144769230769 | 274.992 | 118.064901 | Organic acids and derivatives | POS |
| 51 | Prostaglandin I2 | 0.993668615384615 | 107.291 | 353.2281562 | Lipids and lipid-like molecules | POS |
| 52 | Azithromycin | 0.993407615384615 | 145.404 | 749.5257942 | Organic oxygen compounds | POS |
| 53 | PC(22:5(7Z,10Z,13Z,16Z,19Z)/18:3(6Z,9Z,12Z)) | 0.993215076923077 | 58.43695 | 830.5657574 | Lipids and lipid-like molecules | POS |
| 54 | PC(P-18:1(11Z)/20:5(5Z,8Z,11Z,14Z,17Z)) | 0.992982538461538 | 140.742 | 790.5705225 | Lipids and lipid-like molecules | POS |
| 55 | L-prolyl-L-proline | 0.992981307692308 | 433.0115 | 213.1224383 | Organic acids and derivatives | POS |
| 56 | (2R)-2-aminopropanoic acid | 0.992870230769231 | 362.222 | 90.05496193 | Organic acids and derivatives | POS |
| 57 | L-Histidine | 0.992686307692308 | 376.6055 | 155.117022 | Organic acids and derivatives | POS |
| 58 | Creatine | 0.992432230769231 | 365.7485 | 132.0760684 | Organic acids and derivatives | POS |
| 59 | N-Acetylmuramoyl-Ala | 0.991993384615385 | 364.901 | 365.1550424 | Organic oxygen compounds | POS |
| 60 | Decanoylcarnitine | 0.991388538461538 | 213.126 | 316.2465354 | Lipids and lipid-like molecules | POS |
| 61 | Betaine aldehyde | 0.991276076923077 | 373.406 | 102.091314 | Organic nitrogen compounds | POS |
| 62 | Momordin Ie | 0.990573923076923 | 212.2965 | 897.4756123 | Lipids and lipid-like molecules | POS |
| 63 | Guanine | 0.989840384615384 | 244.341 | 152.0561773 | Organoheterocyclic compounds | POS |
| 64 | (8S,9R,10S,11S,13S,14S,16R,17R)-9-fluoro-11,17-dihydroxy-17-(2-hydroxyacetyl)-10,13,16-trimethyl-6,7,8,11,12,14,15,16-octahydrocyclopenta[a]phenanthren-3-one | 0.989241769230769 | 169.249 | 393.0485921 | Lipids and lipid-like molecules | POS |
| 65 | (2S)-2-amino-4-methylsulfanylbutanoic acid | 0.988976230769231 | 274.107 | 149.0592051 | Organic acids and derivatives | POS |
| 66 | Oleamide | 0.988945615384615 | 77.74605 | 282.277867 | Lipids and lipid-like molecules | POS |
| 67 | Piperidine | 0.988890846153846 | 281.137 | 86.09649874 | Organoheterocyclic compounds | POS |
| 68 | 4-(diaminomethylideneamino)butanoic acid | 0.988862230769231 | 392.594 | 145.096687 | Organic acids and derivatives | POS |
| 69 | Lutein | 0.988506538461538 | 56.5831 | 568.3858361 | Lipids and lipid-like molecules | POS |
| 70 | 2-Pyrrolidineacetic acid | 0.987781846153846 | 218.6335 | 130.0607553 | Organoheterocyclic compounds | POS |
| 71 | PC(22:6(4Z,7Z,10Z,13Z,16Z,19Z)/16:1(9Z)) | 0.986843230769231 | 151.5405 | 803.5546487 | Lipids and lipid-like molecules | POS |
| 72 | PC(18:3(9Z,12Z,15Z)/18:2(9Z,12Z)) | 0.986813846153846 | 57.5513 | 779.5742883 | Lipids and lipid-like molecules | POS |
| 73 | alpha-Tocopherol succinate | 0.986750846153846 | 52.1428 | 531.3682325 | Lipids and lipid-like molecules | POS |
| 74 | Vigabatrin | 0.986541846153846 | 56.2149 | 130.0606175 | Organic acids and derivatives | POS |
| 75 | 5a-Cholesta-8,24-dien-3-one | 0.986074615384615 | 30.9473 | 383.328879 | Lipids and lipid-like molecules | POS |
| 76 | Pyrrolidonecarboxylic acid | 0.986072923076923 | 381.925 | 129.9564183 | Organic acids and derivatives | POS |
| 77 | PS(18:0/20:4(5Z,8Z,11Z,14Z)) | 0.986062846153846 | 195.474 | 812.6699201 | Lipids and lipid-like molecules | POS |
| 78 | Thiamine | 0.985993923076923 | 398.824 | 265.1209863 | Organoheterocyclic compounds | POS |
| 79 | L-Asparagine | 0.985968 | 390.828 | 133.0603315 | Organic acids and derivatives | POS |
| 80 | (3R)-3-hydroxy-4-(trimethylazaniumyl)butanoate | 0.985572 | 419.4495 | 163.0787551 | Organic nitrogen compounds | POS |
| 81 | 2-(1H-imidazol-5-yl)ethanamine | 0.985393846153846 | 410.4785 | 112.0866222 | Organic nitrogen compounds | POS |
| 82 | Acetylhydrazine | 0.985262615384615 | 339.8655 | 75.0555308 | Organic acids and derivatives | POS |
| 83 | Stachyose | 0.985165923076923 | 507.959 | 689.2084843 | Organic oxygen compounds | POS |
| 84 | PE(16:0/22:6(4Z,7Z,10Z,13Z,16Z,19Z)) | 0.984953384615385 | 153.359 | 764.5173945 | Lipids and lipid-like molecules | POS |
| 85 | PE(20:2(11Z,14Z)/20:2(11Z,14Z)) | 0.984741 | 150.985 | 796.58058 | Lipids and lipid-like molecules | POS |
| 86 | Mulberrofuran A | 0.984500769230769 | 75.9726 | 393.2078915 | Phenylpropanoids and polyketides | POS |
| 87 | apo-[3-methylcrotonoyl-CoA:carbon-dioxide ligase (ADP-forming)] | 0.984486769230769 | 308.046 | 174.0810636 | Organic acids and derivatives | POS |
| 88 | LysoPE(16:0/0:0) | 0.984449076923077 | 223.0245 | 454.2908979 | Lipids and lipid-like molecules | POS |
| 89 | Capsicoside C2 | 0.983522692307692 | 214.968 | 873.4756734 | Lipids and lipid-like molecules | POS |
| 90 | Prolyl-Alanine | 0.983050923076923 | 381.9185 | 186.9947463 | Organic acids and derivatives | POS |
| 91 | (2R,3S,4S,5R,6R)-2-(hydroxymethyl)-6-[(2R,3R,4S,5S,6R)-3,4,5-trihydroxy-6-(hydroxymethyl)oxan-2-yl]oxyoxane-3,4,5-triol | 0.982785076923077 | 414.9265 | 364.1268534 | Organic oxygen compounds | POS |
| 92 | Indole-3-carboxylic acid | 0.982685615384615 | 449.661 | 253.164665 | Organoheterocyclic compounds | POS |
| 93 | Clindamycin | 0.98256 | 58.4539 | 424.7754696 | Organic acids and derivatives | POS |
| 94 | Fluvoxamine | 0.982162153846154 | 157.8145 | 319.301912 | Benzenoids | POS |
| 95 | Methylpyrazine | 0.982086384615385 | 410.443 | 95.06033323 | Organoheterocyclic compounds | POS |
| 96 | Nicotine | 0.982021307692308 | 57.617 | 164.0812711 | Organoheterocyclic compounds | POS |
| 97 | 8-Hydroxyadenine | 0.981867923076923 | 194.8505 | 152.0561123 | Organoheterocyclic compounds | POS |
| 98 | Nervonyl carnitine | 0.980651615384615 | 33.6269 | 103.0541673 | Organic nitrogen compounds | POS |
| 99 | Aminoacetone | 0.980237153846154 | 367.533 | 74.06028407 | Organic oxygen compounds | POS |
| 100 | PS(16:0/16:0) | 0.980176538461538 | 37.9627 | 735.5688622 | Lipids and lipid-like molecules | POS |
| 101 | PS(22:4(7Z,10Z,13Z,16Z)/18:1(9Z)) | 0.979063153846154 | 58.41425 | 837.6164268 | Lipids and lipid-like molecules | POS |
| 102 | PE-NMe2(18:0/18:0) | 0.978900692307692 | 57.3741 | 776.5543015 | Lipids and lipid-like molecules | POS |
| 103 | 11-Dehydrocorticosterone | 0.978840692307692 | 8.335415 | 344.2261674 | Lipids and lipid-like molecules | POS |
| 104 | 2-(2-hydroxyethylamino)ethanol | 0.978181153846154 | 377.403 | 107.0602259 | Organic nitrogen compounds | POS |
| 105 | Hovenoside I | 0.977317230769231 | 213.162 | 899.49077 | Lipids and lipid-like molecules | POS |
| 106 | 9-fluoro-11,17-dihydroxy-17-(2-hydroxyacetyl)-10,13,16-trimethyl-6,7,8,11,12,14,15,16-octahydrocyclopenta[a]phenanthren-3-one | 0.977244461538461 | 231.859 | 393.2075525 | Lipids and lipid-like molecules | POS |
| 107 | Camelinin | 0.977146307692307 | 411.633 | 262.1268974 | Organosulfur compounds | POS |
| 108 | 4-(Methylthio)-1-butanol | 0.977064461538461 | 346.798 | 120.9659207 | Organosulfur compounds | POS |
| 109 | PS(16:0/15:0) | 0.977058 | 78.3657 | 721.5536079 | Lipids and lipid-like molecules | POS |
| 110 | Dimethyl dialkyl ammonium chloride | 0.975610846153846 | 52.285 | 305.1554864 | Organonitrogen compounds | POS |
| 111 | Linoleamide | 0.975492615384615 | 164.8035 | 279.7194368 | Lipids and lipid-like molecules | POS |
| 112 | Proline betaine | 0.974926230769231 | 275.039 | 144.0802833 | Organic acids and derivatives | POS |
| 113 | (1S,2S,4R,8S,9S,11S,12S,13R,19S)-19-fluoro-11-hydroxy-8-(2-hydroxyacetyl)-6,6,9,13-tetramethyl-5,7-dioxapentacyclo[10.8.0.02,9.04,8.013,18]icos-17-en-16-one | 0.974858384615384 | 141.606 | 436.7757889 | Lipids and lipid-like molecules | POS |
| 114 | PC(18:4(6Z,9Z,12Z,15Z)/P-16:0) | 0.974137076923077 | 95.6625 | 738.5435178 | Lipids and lipid-like molecules | POS |
| 115 | 2-aminoethanesulfinic acid | 0.973350307692308 | 346.855 | 109.9165366 | Organic acids and derivatives | POS |
| 116 | Arecaidine | 0.972885153846154 | 222.868 | 141.1380309 | Alkaloids and derivatives | POS |
| 117 | 5-Hydroxy-7-methoxy-2-tritriacontyl-4H-1-benzopyran-4-one | 0.972386769230769 | 210.5035 | 655.5640255 | Organoheterocyclic compounds | POS |
| 118 | Alanyl-Valine | 0.971911307692307 | 46.7869 | 189.1226664 | Organic acids and derivatives | POS |
| 119 | Neocretanin | 0.971044846153846 | 346.838 | 468.7296997 | Organic oxygen compounds | POS |
| 120 | PE(20:0/18:4(6Z,9Z,12Z,15Z)) | 0.970723538461538 | 152.558 | 768.5478926 | Lipids and lipid-like molecules | POS |
| 121 | N-Hexadecanoylpyrrolidine | 0.970203769230769 | 72.5951 | 310.3087002 | Organoheterocyclic compounds | POS |
| 122 | Capsianoside VI | 0.970117538461538 | 210.5145 | 923.4942367 | Lipids and lipid-like molecules | POS |
| 123 | N,N-dimethylmethanamine oxide | 0.968965538461538 | 360.399 | 76.0759126 | Organic nitrogen compounds | POS |
| 124 | 7-Chloro-6-demethylcepharadione B | 0.968694 | 206.13 | 341.9903756 | Alkaloids and derivatives | POS |
| 125 | 5-aminopentanoic acid | 0.968303153846154 | 402.673 | 118.0859713 | Organic acids and derivatives | POS |
| 126 | Arachidonyl carnitine | 0.968068923076923 | 184.3775 | 504.4036664 | Lipids and lipid-like molecules | POS |
| 127 | p-Anisic acid | 0.966998 | 244.3575 | 153.0594132 | Benzenoids | POS |
| 128 | Diisopropyl sulfide | 0.966346153846154 | 275.237 | 119.0682808 | Organosulfur compounds | POS |
| 129 | PC(20:5(5Z,8Z,11Z,14Z,17Z)/P-18:1(11Z)) | 0.966334538461538 | 37.9482 | 789.6161794 | Lipids and lipid-like molecules | POS |
| 130 | SM(d18:1/24:1(15Z)) | 0.965819153846154 | 209.644 | 813.5433918 | Lipids and lipid-like molecules | POS |
| 131 | 4-(trimethylazaniumyl)butanoate | 0.965758384615385 | 395.0605 | 146.1169067 | Lipids and lipid-like molecules | POS |
| 132 | PC(22:6(4Z,7Z,10Z,13Z,16Z,19Z)/P-18:1(11Z)) | 0.964206461538461 | 61.99725 | 815.631201 | Lipids and lipid-like molecules | POS |
| 133 | PE(20:0/20:2(11Z,14Z)) | 0.963787769230769 | 62.91215 | 800.6123765 | Lipids and lipid-like molecules | POS |
| 134 | 1,7-Dimethylguanosine | 0.963517230769231 | 189.1585 | 311.9718523 | Nucleosides, nucleotides, and analogues | POS |
| 135 | Symmetric dimethylarginine | 0.963101538461538 | 514.5695 | 203.1495048 | Organic acids and derivatives | POS |
| 136 | Solanidine | 0.962375769230769 | 72.78895 | 398.7598302 | Lipids and lipid-like molecules | POS |
| 137 | N-Acetylcadaverine | 0.962359384615385 | 335.03 | 145.1330206 | Organic acids and derivatives | POS |
| 138 | Coriandrinonediol | 0.962223615384615 | 27.25635 | 460.2921749 | lipids and lipid-like molecules | POS |
| 139 | (2S)-5-amino-5-oxo-2-[(2-phenylacetyl)amino]pentanoic acid | 0.962061692307692 | 236.3725 | 265.1166382 | Organic acids and derivatives | POS |
| 140 | Actinidine | 0.961826 | 214.18 | 148.0420739 | Organoheterocyclic compounds | POS |
| 141 | 2-amino-9-[(2R,3R,4S,5R)-3,4-dihydroxy-5-(hydroxymethyl)oxolan-2-yl]-1H-purin-6-one | 0.960772846153846 | 280.3205 | 284.0976825 | Nucleosides, nucleotides, and analogues | POS |
| 142 | N-Acetylhistidine | 0.960477461538461 | 360.4 | 198.0867465 | Organic acids and derivatives | POS |
| 143 | Hoduloside VII | 0.959465538461538 | 209.6885 | 930.6021448 | Lipids and lipid-like molecules | POS |
| 144 | Trimethylamine N-oxide | 0.959351 | 343.2995 | 76.07590492 | Organic nitrogen compounds | POS |
| 145 | N-methyl-L-glutamic Acid | 0.959023230769231 | 402.5445 | 161.135944 | Organic acids and derivatives | POS |
| 146 | Dimethylethanolamine | 0.958789461538461 | 285.7455 | 90.09138238 | Organonitrogen compounds | POS |
| 147 | 2-O-(5,8,11,14,17-Eicosapentaenoyl)-1-O-hexadecylglycero-3-phosphocholine | 0.957833615384615 | 57.9639 | 766.5720043 | Lipids and lipid-like molecules | POS |
| 148 | Helipyrone | 0.957819153846154 | 156.354 | 320.2931787 | Organoheterocyclic compounds | POS |
| 149 | PE(P-18:1(11Z)/22:5(4Z,7Z,10Z,13Z,16Z)) | 0.957465384615385 | 154.3075 | 775.5994698 | Lipids and lipid-like molecules | POS |
| 150 | Dehydrophytosphingosine | 0.956635307692308 | 142.7605 | 316.2827851 | Organic nitrogen compounds | POS |
| 151 | (2S)-2-amino-5-[[(2R)-1-(carboxymethylamino)-1-oxo-3-sulfanylpropan-2-yl]amino]-5-oxopentanoic acid | 0.956269 | 381.089 | 307.9689799 | Organic acids and derivatives | POS |
| 152 | Albanol B | 0.956047076923077 | 355.844 | 559.0738705 | Phenylpropanoids and polyketides | POS |
| 153 | L-Hyoscyamine | 0.955034 | 224.742 | 289.2186368 | Alkaloids and derivatives | POS |
| 154 | [(2S,3S,5S,8R,9S,10S,13S,14S,16S,17R)-17-acetyloxy-10,13-dimethyl-16-(1-methylpiperidin-1-ium-1-yl)-2-piperidin-1-yl-2,3,4,5,6,7,8,9,11,12,14,15,16,17-tetradecahydro-1H-cyclopenta[a]phenanthren-3-yl] acetate | 0.954836 | 164.786 | 557.4301501 | Lipids and lipid-like molecules | POS |
| 155 | gamma-Aminobutyric acid | 0.952872692307692 | 382.7255 | 103.0945497 | Organic acids and derivatives | POS |
| 156 | 2,6-Toluenediamine | 0.952743076923077 | 49.4295 | 123.0913358 | Benzenoids | POS |
| 157 | LysoPE(0:0/20:4(5Z,8Z,11Z,14Z)) | 0.952465461538462 | 215.187 | 502.2912798 | Lipids and lipid-like molecules | POS |
| 158 | PC(22:6(4Z,7Z,10Z,13Z,16Z,19Z)/22:5(4Z,7Z,10Z,13Z,16Z)) | 0.952334692307692 | 149.772 | 880.5326542 | Lipids and lipid-like molecules | POS |
| 159 | PE(20:3(5Z,8Z,11Z)/P-18:1(11Z)) | 0.950597076923077 | 82.2637 | 752.5536661 | Lipids and lipid-like molecules | POS |
| 160 | 1-(5Z,8Z,11Z,14Z-eicosatetraenoyl)-sn-glycero-3-phosphate | 0.950138923076923 | 186.837 | 458.4099964 | Lipids and lipid-like molecules | POS |
| 161 | PC(22:6(4Z,7Z,10Z,13Z,16Z,19Z)/18:4(6Z,9Z,12Z,15Z)) | 0.949680230769231 | 181.11 | 826.0731709 | Lipids and lipid-like molecules | POS |
| 162 | (2S)-2-amino-5-(carbamoylamino)pentanoic acid | 0.949480615384615 | 428.748 | 177.0321806 | Organic acids and derivatives | POS |
| 163 | Guanidoacetic acid | 0.949353538461538 | 359.475 | 117.1020215 | Organic acids and derivatives | POS |
| 164 | L-beta-aspartyl-L-phenylalanine | 0.949240846153846 | 346.791 | 280.9480048 | Organic acids and derivatives | POS |
| 165 | Oxolan-3-one | 0.948052230769231 | 219.4225 | 87.04410635 | Organoheterocyclic compounds | POS |
| 166 | piperidine-2-carboxylic acid | 0.946906923076923 | 540.3685 | 129.1018109 | Organic acids and derivatives | POS |
| 167 | L-alpha-Aspartyl-L-hydroxyproline | 0.946888076923077 | 311.775 | 247.024248 | Organic acids and derivatives | POS |
| 168 | [(2R)-2-acetyloxy-3-carboxypropyl]-trimethylazanium | 0.946809769230769 | 324.229 | 204.1218172 | Lipids and lipid-like molecules | POS |
| 169 | PC(16:0/16:0) | 0.946529076923077 | 168.3395 | 733.9934322 | Lipids and lipid-like molecules | POS |
| 170 | Valyl-Lysine | 0.944131769230769 | 581.416 | 246.1798191 | Organic acids and derivatives | POS |
| 171 | PE(18:4(6Z,9Z,12Z,15Z)/P-18:1(11Z)) | 0.943881846153846 | 77.9921 | 722.5577313 | Lipids and lipid-like molecules | POS |
| 172 | PA(22:6(4Z,7Z,10Z,13Z,16Z,19Z)/20:2(11Z,14Z)) | 0.942941846153846 | 203.9635 | 773.5071224 | Lipids and lipid-like molecules | POS |
| 173 | LysoPE(0:0/18:3(6Z,9Z,12Z)) | 0.942700615384615 | 194.945 | 475.301624 | Lipids and lipid-like molecules | POS |
| 174 | Oxepahyperforin | 0.941898461538461 | 25.49 | 553.3120933 | Lipids and lipid-like molecules | POS |
| 175 | PE(22:5(4Z,7Z,10Z,13Z,16Z)/18:0) | 0.941244230769231 | 64.78985 | 794.5637692 | Lipids and lipid-like molecules | POS |
| 176 | (1R,2R,3S,5S)-3-hydroxy-8-methyl-8-azabicyclo[3.2.1]octane-2-carboxylic acid | 0.941056923076923 | 318.81 | 186.1055661 | Alkaloids and derivatives | POS |
| 177 | PC(16:1(9Z)/18:4(6Z,9Z,12Z,15Z)) | 0.939194846153846 | 58.8578 | 751.5423633 | Lipids and lipid-like molecules | POS |
| 178 | 3-Hydroxyisovalerylcarnitine | 0.937662384615385 | 324.233 | 262.1634759 | Lipids and lipid-like molecules | POS |
| 179 | PC(22:5(4Z,7Z,10Z,13Z,16Z)/14:0) | 0.937494384615385 | 197.33 | 779.6026534 | Lipids and lipid-like molecules | POS |
| 180 | 1-Methylhistidine | 0.937168461538461 | 399.7765 | 169.1328615 | Organic acids and derivatives | POS |
| 181 | 3-Acetyl-2,7-naphthyridine | 0.936313538461538 | 40.60285 | 172.1325582 | Organoheterocyclic compounds | POS |
| 182 | D-Proline | 0.934164692307692 | 380.1065 | 116.0815771 | Organic acids and derivatives | POS |
| 183 | (2S)-2-amino-5-[[(1S)-4-amino-1-carboxy-4-oxobutyl]amino]-5-oxopentanoic acid | 0.933833846153846 | 430.993 | 276.0018305 | Organic acids and derivatives | POS |
| 184 | PE(22:4(7Z,10Z,13Z,16Z)/14:1(9Z)) | 0.933618461538461 | 162.129 | 738.5025415 | Lipids and lipid-like molecules | POS |
| 185 | Ropivacaine | 0.933567692307692 | 58.9528 | 275.275792 | Organic acids and derivatives | POS |
| 186 | [[(2R,3S,4R,5R)-5-(6-aminopurin-9-yl)-3,4-dihydroxyoxolan-2-yl]methoxy-hydroxyphosphoryl] [(2S,3R,4S,5S)-5-(3-carbamoylpyridin-1-ium-1-yl)-3,4-dihydroxyoxolan-2-yl]methyl phosphate | 0.933235692307692 | 447.83 | 664.1155367 | Nucleosides, nucleotides, and analogues | POS |
| 187 | 2-Dodecylbenzenesulfonic acid | 0.931040923076923 | 176.969 | 328.000594 | Benzenoids | POS |
| 188 | PC(20:4(8Z,11Z,14Z,17Z)/P-18:0) | 0.931023615384615 | 155.012 | 794.06815 | Lipids and lipid-like molecules | POS |
| 189 | 1-Methyladenosine | 0.929109461538461 | 142.757 | 281.2653692 | Nucleosides, nucleotides, and analogues | POS |
| 190 | 4,4-Dimethoxy-2-butanone | 0.928822923076923 | 182.1485 | 133.08553 | Organic oxygen compounds | POS |
| 191 | PE(20:4(5Z,8Z,11Z,14Z)/P-18:1(11Z)) | 0.928292384615385 | 73.51 | 749.5816787 | Lipids and lipid-like molecules | POS |
| 192 | PE(16:0/20:4(5Z,8Z,11Z,14Z)) | 0.927722 | 59.80805 | 739.5442998 | Lipids and lipid-like molecules | POS |
| 193 | PE(22:5(4Z,7Z,10Z,13Z,16Z)/14:0) | 0.926255538461539 | 46.7709 | 737.5205392 | Lipids and lipid-like molecules | POS |
| 194 | Melphalan | 0.925559384615385 | 295.608 | 305.1552795 | Organic acids and derivatives | POS |
| 195 | Arginyl-Valine | 0.924056153846154 | 435.951 | 274.1017635 | Organic acids and derivatives | POS |
| 196 | PS(22:4(7Z,10Z,13Z,16Z)/18:3(6Z,9Z,12Z)) | 0.923706153846154 | 208.798 | 834.5244209 | Lipids and lipid-like molecules | POS |
| 197 | LysoPC(P-18:1(9Z)) | 0.923705615384615 | 184.1835 | 506.4195549 | Lipids and lipid-like molecules | POS |
| 198 | 4-(Nitrosoamino)-1-(3-pyridinyl)-1-butanone | 0.922848153846154 | 46.82315 | 194.1531847 | Organic oxygen compounds | POS |
| 199 | Trimethylaminoacetone | 0.920811769230769 | 230.143 | 116.1067831 | Organic oxygen compounds | POS |
| 200 | Cytarabine | 0.920263923076923 | 256.394 | 244.0914532 | Nucleosides, nucleotides, and analogues | POS |
| 201 | PC(18:4(6Z,9Z,12Z,15Z)/18:2(9Z,12Z)) | 0.920070846153846 | 141.891 | 777.5571089 | Lipids and lipid-like molecules | POS |
| 202 | N-Cyclopropyl-trans-2-cis-6-nonadienamide | 0.919547076923077 | 596.115 | 194.1532029 | Lipids and lipid-like molecules | POS |
| 203 | DG(20:3(8Z,11Z,14Z)/16:1(9Z)/0:0) | 0.918342923076923 | 30.87715 | 617.511647 | Lipids and lipid-like molecules | POS |
| 204 | (2S)-N-methyl-1-phenylpropan-2-amine | 0.917780307692308 | 202.603 | 150.1270998 | Benzenoids | POS |
| 205 | PC(22:6(4Z,7Z,10Z,13Z,16Z,19Z)/18:3(6Z,9Z,12Z)) | 0.917729153846154 | 147.243 | 828.5498627 | Lipids and lipid-like molecules | POS |
| 206 | Formiminoglutamic acid | 0.916976076923077 | 438.367 | 174.0867378 | Organic acids and derivatives | POS |
| 207 | PE(20:4(5Z,8Z,11Z,14Z)/P-18:0) | 0.915322076923077 | 199.395 | 751.5685685 | Lipids and lipid-like molecules | POS |
| 208 | Heptane-1-thiol | 0.913886538461538 | 291.969 | 133.104752 | Organosulfur compounds | POS |
| 209 | Allysine | 0.913601538461539 | 319.782 | 146.0806445 | Organic acids and derivatives | POS |
| 210 | SM(d18:1/12:0) | 0.913221692307692 | 206.129 | 647.5097713 | Lipids and lipid-like molecules | POS |
| 211 | DG(22:4(7Z,10Z,13Z,16Z)/14:1(9Z)/0:0) | 0.910092230769231 | 54.8469 | 615.4532333 | Lipids and lipid-like molecules | POS |
| 212 | Methyl 3-(2,3-dihydroxy-3-methylbutyl)-4-hydroxybenzoate | 0.908791153846154 | 84.2695 | 255.2497508 | Benzenoids | POS |
| 213 | 1-Methyl 2-galloylgalactarate | 0.908756076923077 | 438.284 | 376.2075861 | Organic oxygen compounds | POS |
| 214 | PE(18:3(9Z,12Z,15Z)/P-18:0) | 0.907384615384615 | 148.9275 | 726.5363202 | Lipids and lipid-like molecules | POS |
| 215 | Glycylalanylprolylmethionylphenylalanylvalinamide | 0.905432461538461 | 207.059 | 620.3214121 | Organic acids and derivatives | POS |
| 216 | Apiumoside | 0.901560769230769 | 470.012 | 570.3225001 | Phenylpropanoids and polyketides | POS |
| 217 | Bosentan | 0.901291153846154 | 280.476 | 552.1869584 | Organoheterocyclic compounds | POS |
| 218 | PE(18:2(9Z,12Z)/P-16:0) | 0.901229384615385 | 73.4965 | 700.9652737 | Lipids and lipid-like molecules | POS |
| 219 | 2-[bis(2-hydroxyethyl)amino]ethanol | 0.900353230769231 | 202.382 | 149.1148918 | Organonitrogen compounds | POS |
| 220 | PC(22:6(4Z,7Z,10Z,13Z,16Z,19Z)/22:6(4Z,7Z,10Z,13Z,16Z,19Z)) | 0.898411846153846 | 169.3 | 878.0628577 | Lipids and lipid-like molecules | POS |
| 221 | PC(22:6(4Z,7Z,10Z,13Z,16Z,19Z)/20:3(5Z,8Z,11Z)) | 0.897273076923077 | 69.956 | 856.5324348 | Lipids and lipid-like molecules | POS |
| 222 | (2S)-2-amino-6-(diaminomethylideneamino)hexanoic acid | 0.896715615384615 | 537.4215 | 189.1338689 | Organic acids and derivatives | POS |
| 223 | Netilmicin | 0.895663769230769 | 88.5517 | 476.3056982 | Organic oxygen compounds | POS |
| 224 | PE(18:1(11Z)/P-18:1(11Z)) | 0.894847076923077 | 38.8455 | 728.556288 | Lipids and lipid-like molecules | POS |
| 225 | (E)-3-(3,4,5-trimethoxyphenyl)prop-2-enoic acid | 0.894736538461538 | 381.956 | 276.9130578 | Phenylpropanoids and polyketides | POS |
| 226 | (3S)-3-amino-4-[[(2S)-1-methoxy-1-oxo-3-phenylpropan-2-yl]amino]-4-oxobutanoic acid | 0.892895076923077 | 346.8015 | 294.9279295 | Organic acids and derivatives | POS |
| 227 | Linoleyl carnitine | 0.885177615384615 | 215.071 | 424.2807048 | Lipids and lipid-like molecules | POS |
| 228 | dTDP-4-acetamido-4,6-dideoxy-D-galactose | 0.883593076923077 | 452.655 | 591.1223764 | Nucleosides, nucleotides, and analogues | POS |
| 229 | PC(22:4(7Z,10Z,13Z,16Z)/15:0) | 0.882437769230769 | 57.73815 | 795.6048312 | Lipids and lipid-like molecules | POS |
| 230 | 2-Azetidinecarboxylic acid | 0.882320461538462 | 387.299 | 101.0708493 | Organic acids and derivatives | POS |
| 231 | 2-Ethyl-2-Hydroxybutyric acid | 0.881061615384615 | 269.264 | 133.0855217 | Lipids and lipid-like molecules | POS |
| 232 | LysoPC(15:0) | 0.880821923076923 | 207.019 | 481.3472392 | Lipids and lipid-like molecules | POS |
| 233 | beta-Farnesene | 0.877658153846154 | 39.7863 | 206.0766938 | Lipids and lipid-like molecules | POS |
| 234 | Butyrylcarnitine | 0.876641230769231 | 311.746 | 232.0405148 | Lipids and lipid-like molecules | POS |
| 235 | N-Palmitoylsphingosine | 0.876034384615385 | 54.6399 | 537.8603498 | Lipids and lipid-like molecules | POS |
| 236 | 4-(Methylnitrosamino)-1-(3-pyridyl)-1-butanone | 0.875556538461538 | 493.363 | 207.9712754 | Organic oxygen compounds | POS |
| 237 | LysoPC(20:4(5Z,8Z,11Z,14Z)) | 0.875189076923077 | 181.546 | 545.0251741 | Lipids and lipid-like molecules | POS |
| 238 | PC(22:5(4Z,7Z,10Z,13Z,16Z)/15:0) | 0.872671615384615 | 56.8026 | 793.5912699 | Lipids and lipid-like molecules | POS |
| 239 | PC(P-16:0/18:1(11Z)) | 0.871285538461538 | 72.8155 | 745.5557372 | Lipids and lipid-like molecules | POS |
| 240 | N2-Methylguanine | 0.871013692307692 | 196.4535 | 166.0718291 | Organoheterocyclic compounds | POS |
| 241 | PS(18:1(9Z)/20:4(5Z,8Z,11Z,14Z)) | 0.869215230769231 | 195.577 | 809.6506536 | Lipids and lipid-like molecules | POS |
| 242 | (2S)-2,6-diaminohexanoic acid | 0.868753230769231 | 508.177 | 146.1281805 | Organic acids and derivatives | POS |
| 243 | N-Lactoyl ethanolamine | 0.868722846153846 | 19.68395 | 135.0316069 | Organonitrogen compounds | POS |
| 244 | PC(18:3(9Z,12Z,15Z)/16:1(9Z)) | 0.865457153846154 | 199.9605 | 753.5847439 | Lipids and lipid-like molecules | POS |
| 245 | PC(P-16:0/18:4(6Z,9Z,12Z,15Z)) | 0.865130923076923 | 38.3829 | 738.5409085 | Lipids and lipid-like molecules | POS |
| 246 | 2-Phenoxyethyl isobutyrate | 0.865029461538461 | 46.6448 | 209.1024654 | Benzenoids | POS |
| 247 | 1-aminocyclopentane-1-carboxylic acid | 0.864315076923077 | 652.183 | 129.956538 | Organic acids and derivatives | POS |
| 248 | PE(20:5(5Z,8Z,11Z,14Z,17Z)/P-16:0) | 0.864019538461538 | 161.084 | 721.5533115 | Lipids and lipid-like molecules | POS |
| 249 | PC(18:3(6Z,9Z,12Z)/P-18:0) | 0.863931923076923 | 59.27035 | 768.586026 | Lipids and lipid-like molecules | POS |
| 250 | Stigmastane-3,6-dione | 0.861580461538462 | 30.8514 | 429.371458 | Lipids and lipid-like molecules | POS |
| 251 | PS(14:0/15:0) | 0.858373 | 86.78955 | 693.5221257 | Lipids and lipid-like molecules | POS |
| 252 | Zymonic acid | 0.858318153846154 | 175.467 | 159.0269299 | Organoheterocyclic compounds | POS |
| 253 | PS(18:0/22:6(4Z,7Z,10Z,13Z,16Z,19Z)) | 0.857055538461538 | 208.747 | 836.5388507 | Lipids and lipid-like molecules | POS |
| 254 | LysoPC(22:6(4Z,7Z,10Z,13Z,16Z,19Z)) | 0.856680846153846 | 165.008 | 568.1175274 | Lipids and lipid-like molecules | POS |
| 255 | 2-amino-4-methylsulfanylbutanoic acid | 0.856199153846154 | 332.3445 | 150.0577481 | Organic acids and derivatives | POS |
| 256 | 3-Methylglutarylcarnitine | 0.856029230769231 | 400.66 | 290.1581751 | Lipids and lipid-like molecules | POS |
| 257 | 2-O-Methylcytosine | 0.855893461538461 | 98.379 | 126.0739196 | Organoheterocyclic compounds | POS |
| 258 | Lactosylceramide (d18:1/16:0) | 0.854397384615385 | 206.1195 | 862.622648 | Lipids and lipid-like molecules | POS |
| 259 | PE(20:5(5Z,8Z,11Z,14Z,17Z)/14:0) | 0.853513 | 162.197 | 709.5450583 | Lipids and lipid-like molecules | POS |
| 260 | N-Methylnicotinamide | 0.853496384615385 | 46.67695 | 137.0704813 | Organoheterocyclic compounds | POS |
| 261 | PE(16:0/18:3(9Z,12Z,15Z)) | 0.852985923076923 | 169.7685 | 714.0613689 | Lipids and lipid-like molecules | POS |
| 262 | Cyclotetradecane | 0.852706230769231 | 67.4295 | 227.1743557 | Hydrocarbons | POS |
| 263 | Molybdopterin precursor Z | 0.851200538461538 | 501.7375 | 364.7005965 | Organoheterocyclic compounds | POS |
| 264 | 6-Hydroxy-1H-indole-3-acetamide | 0.850527384615385 | 46.7276 | 191.0807232 | Organoheterocyclic compounds | POS |
| 265 | 4-Trimethylammoniobutanal | 0.850440769230769 | 269.463 | 130.1221905 | Organic oxygen compounds | POS |
| 266 | Fluconazole | 0.850383846153846 | 381.967 | 306.1585413 | Benzenoids | POS |
| 267 | piperazine | 0.848044692307692 | 350.33 | 86.90872461 | Organoheterocyclic compounds | POS |
| 268 | Prolyl-Valine | 0.847774615384615 | 317.014 | 215.0952606 | Organic acids and derivatives | POS |
| 269 | 1,9-Nonanedithiol | 0.845392538461538 | 283.96 | 193.1076071 | Organosulfur compounds | POS |
| 270 | 3-O-ethyl 5-O-methyl 2-(2-aminoethoxymethyl)-4-(2-chlorophenyl)-6-methyl-1,4-dihydropyridine-3,5-dicarboxylate | 0.844442769230769 | 110.844 | 409.1506425 | Organoheterocyclic compounds | POS |
| 271 | 2-[methyl-(N'-phosphonocarbamimidoyl)amino]acetic acid | 0.842507461538461 | 452.0455 | 212.0422439 | Organic acids and derivatives | POS |
| 272 | Chlorhexidine | 0.839576 | 330.498 | 505.2099871 | Benzenoids | POS |
| 273 | PC(20:5(5Z,8Z,11Z,14Z,17Z)/20:4(5Z,8Z,11Z,14Z)) | 0.838117230769231 | 59.85005 | 828.5496042 | Lipids and lipid-like molecules | POS |
| 274 | 13-chloro-2-piperidin-4-ylidene-4-azatricyclo[9.4.0.03,8]pentadeca-1(11),3(8),4,6,12,14-hexaene | 0.836166076923077 | 223.911 | 310.1996479 | Organoheterocyclic compounds | POS |
| 275 | Oxidized glutathione | 0.835411692307692 | 481.331 | 613.127956 | Organic acids and derivatives | POS |
| 276 | pyridine-3-carboxamide | 0.832616 | 48.5879 | 122.0709567 | Organoheterocyclic compounds | POS |
| 277 | 3-Methylcytosine | 0.831402076923077 | 199.0885 | 126.0657759 | Organoheterocyclic compounds | POS |
| 278 | (2R)-2-Hydroxy-2-methylbutanenitrile | 0.830905 | 284.8225 | 99.09162142 | Organic oxygen compounds | POS |
| 279 | PE(20:5(5Z,8Z,11Z,14Z,17Z)/P-18:1(11Z)) | 0.828498538461539 | 158.6655 | 747.570589 | Lipids and lipid-like molecules | POS |
| 280 | (-)-Dioxibrassinin | 0.827842076923077 | 345.929 | 268.0501192 | Organic acids and derivatives | POS |
| 281 | 5,7-dihydroxy-2-phenylchromen-4-one | 0.827190538461538 | 330.528 | 254.6076979 | Phenylpropanoids and polyketides | POS |
| 282 | Semilepidinoside B | 0.820686846153846 | 347.619 | 366.1587534 | Organic oxygen compounds | POS |
| 283 | Diepomuricanin A | 0.819456384615385 | 213.129 | 547.3568475 | Lipids and lipid-like molecules | POS |
| 284 | 1-Arachidonoylglycerophosphoinositol | 0.819358923076923 | 257.861 | 621.3009549 | Lipids and lipid-like molecules | POS |
| 285 | L-Cyclo(alanylglycyl) | 0.813805769230769 | 389.044 | 129.0655271 | Organoheterocyclic compounds | POS |
| 286 | 2-Methylbutyroylcarnitine | 0.812864384615385 | 279.427 | 246.0937025 | Lipids and lipid-like molecules | POS |
| 287 | Nonate | 0.812634846153846 | 217.579 | 189.0731049 | Lipids and lipid-like molecules | POS |
| 288 | SM(d18:1/16:0) | 0.812180384615385 | 201.717 | 703.5728421 | Lipids and lipid-like molecules | POS |
| 289 | PC(20:5(5Z,8Z,11Z,14Z,17Z)/20:1(11Z)) | 0.808423307692308 | 144.166 | 834.5957474 | Lipids and lipid-like molecules | POS |
| 290 | 5'-Hydroxymethyl meloxicam | 0.807630461538462 | 248.5625 | 367.9923932 | Organoheterocyclic compounds | POS |
| 291 | (2S,3S,4R)-2-aminooctadecane-1,3,4-triol | 0.804907076923077 | 41.504 | 317.3224987 | Organic nitrogen compounds | POS |
| 292 | LysoPE(22:6(4Z,7Z,10Z,13Z,16Z,19Z)/0:0) | 0.804574153846154 | 181.549 | 526.0176342 | Lipids and lipid-like molecules | POS |
| 293 | 2-Hexaprenyl-6-methoxyphenol | 0.803683230769231 | 165.008 | 533.1092435 | Lipids and lipid-like molecules | POS |
| 294 | Epomusenin A | 0.802412538461538 | 155.067 | 559.5062484 | Lipids and lipid-like molecules | POS |
| 295 | Butyl ethyl malonate | 0.802121692307692 | 291.971 | 189.0174557 | Organic acids and derivatives | POS |
| 296 | PS(22:4(7Z,10Z,13Z,16Z)/20:5(5Z,8Z,11Z,14Z,17Z)) | 0.795329 | 207.901 | 858.5194182 | Lipids and lipid-like molecules | POS |
| 297 | LysoPE(16:1(9Z)/0:0) | 0.794993615384615 | 189.526 | 451.3593506 | Lipids and lipid-like molecules | POS |
| 298 | LysoPC(18:2(9Z,12Z)) | 0.794563769230769 | 188.404 | 520.339001 | Lipids and lipid-like molecules | POS |
| 299 | Heptadecanoyl carnitine | 0.793105230769231 | 192.093 | 414.3563803 | Lipids and lipid-like molecules | POS |
| 300 | PC(18:3(6Z,9Z,12Z)/18:3(6Z,9Z,12Z)) | 0.791348538461539 | 70.6784 | 778.0396757 | Lipids and lipid-like molecules | POS |
| 301 | PC(20:5(5Z,8Z,11Z,14Z,17Z)/15:0) | 0.791013 | 143.657 | 765.5594474 | Lipids and lipid-like molecules | POS |
| 302 | PC(22:5(7Z,10Z,13Z,16Z,19Z)/22:4(7Z,10Z,13Z,16Z)) | 0.790834461538461 | 152.771 | 884.5647498 | Lipids and lipid-like molecules | POS |
| 303 | Inositol 1,3,4-trisphosphate | 0.790124461538461 | 299.107 | 420.9689657 | Organic oxygen compounds | POS |
| 304 | Leucyl-Glycine | 0.789698615384615 | 276.978 | 189.073104 | Organic acids and derivatives | POS |
| 305 | Methylimidazoleacetic acid | 0.788973384615384 | 317.91 | 141.0652908 | Organoheterocyclic compounds | POS |
| 306 | Linoleoyl ethanolamide | 0.787111692307692 | 33.52485 | 324.2879402 | Organic nitrogen compounds | POS |
| 307 | PE(P-18:1(9Z)/18:1(9Z)) | 0.782720076923077 | 151.569 | 728.5553351 | Lipids and lipid-like molecules | POS |
| 308 | Cervonyl carnitine | 0.780812 | 212.271 | 471.3858792 | Lipids and lipid-like molecules | POS |
| 309 | Hydroxyprolyl-Asparagine | 0.778361538461538 | 122.38 | 245.2656189 | Organic acids and derivatives | POS |
| 310 | methyl (2S)-2-amino-3-(4-hydroxyphenyl)propanoate | 0.777812846153846 | 47.9878 | 195.1219474 | Organic acids and derivatives | POS |
| 311 | [12]-Gingerol | 0.777113769230769 | 196.553 | 396.3089329 | Benzenoids | POS |
| 312 | PC(P-18:1(9Z)/16:1(9Z)) | 0.776694769230769 | 150.3725 | 742.5716748 | Lipids and lipid-like molecules | POS |
| 313 | N1,N12-Diacetylspermine | 0.776693076923077 | 311.865 | 287.0467286 | Organic acids and derivatives | POS |
| 314 | Acetone cyanohydrin | 0.773669461538462 | 72.40225 | 86.06009138 | Organic oxygen compounds | POS |
| 315 | PS(20:4(5Z,8Z,11Z,14Z)/20:2(11Z,14Z)) | 0.770604692307692 | 208.791 | 837.5434281 | Lipids and lipid-like molecules | POS |
| 316 | Valyl-Phenylalanine | 0.767130615384615 | 199.959 | 265.1533234 | Organic acids and derivatives | POS |
| 317 | LysoPE(22:5(4Z,7Z,10Z,13Z,16Z)/0:0) | 0.766307 | 213.212 | 527.3787669 | Lipids and lipid-like molecules | POS |
| 318 | Leucyl-Serine | 0.765311307692308 | 315.237 | 219.0965705 | Organic acids and derivatives | POS |
| 319 | Boviquinone 4 | 0.764342461538462 | 63.8489 | 412.7756799 | Lipids and lipid-like molecules | POS |
| 320 | PC(P-18:1(11Z)/22:6(4Z,7Z,10Z,13Z,16Z,19Z)) | 0.763781 | 137.469 | 816.587886 | Lipids and lipid-like molecules | POS |
| 321 | Linoelaidyl carnitine | 0.759782076923077 | 194.796 | 425.0876367 | Lipids and lipid-like molecules | POS |
| 322 | Perilloside A | 0.758162923076923 | 33.51335 | 315.178377 | Lipids and lipid-like molecules | POS |
| 323 | 8-chloro-6-(2-fluorophenyl)-1-methyl-4H-imidazo[1,5-a][1,4]benzodiazepine | 0.756617384615385 | 33.5586 | 326.3033974 | Organoheterocyclic compounds | POS |
| 324 | LysoPE(22:4(7Z,10Z,13Z,16Z)/0:0) | 0.756121153846154 | 208.7505 | 530.3227412 | Lipids and lipid-like molecules | POS |
| 325 | Isoneotheaflavin | 0.755812923076923 | 438.6415 | 565.1363911 | Phenylpropanoids and polyketides | POS |
| 326 | 7,8-dimethyl-10-[(2S,3S,4R)-2,3,4,5-tetrahydroxypentyl]benzo[g]pteridine-2,4-dione | 0.751104923076923 | 231.0215 | 377.1437033 | Organoheterocyclic compounds | POS |
| 327 | methyl 2-(2-chlorophenyl)-2-(6,7-dihydro-4H-thieno[3,2-c]pyridin-5-yl)acetate | 0.750877461538461 | 457.879 | 322.0684481 | Organic acids and derivatives | POS |
| 328 | Chenodeoxycholic acid glycine conjugate | 0.750595769230769 | 201.716 | 450.2573568 | Lipids and lipid-like molecules | POS |
| 329 | Leucyl-Arginine | 0.750572384615385 | 375.6565 | 288.1912719 | Organic acids and derivatives | POS |
| 330 | PS(20:0/20:4(5Z,8Z,11Z,14Z)) | 0.748987 | 209.506 | 840.5716887 | Lipids and lipid-like molecules | POS |
| 331 | (E)-1-(4-methoxyphenyl)-3-phenylprop-2-en-1-one | 0.748330461538461 | 206.054 | 238.9859965 | Phenylpropanoids and polyketides | POS |
| 332 | LysoPE(0:0/20:5(5Z,8Z,11Z,14Z,17Z)) | 0.747856769230769 | 216.7625 | 499.3471621 | Lipids and lipid-like molecules | POS |
| 333 | Spirolide D | 0.747584692307692 | 37.9482 | 707.5396104 | Organoheterocyclic compounds | POS |
| 334 | PC(22:6(4Z,7Z,10Z,13Z,16Z,19Z)/20:1(11Z)) | 0.747089076923077 | 141.07 | 860.6104236 | Lipids and lipid-like molecules | POS |
| 335 | 2-aminoacetic acid | 0.744212076923077 | 379.194 | 76.03954515 | Organic acids and derivatives | POS |
| 336 | beta-Cryptoxanthin | 0.743648230769231 | 25.5608 | 552.308195 | Lipids and lipid-like molecules | POS |
| 337 | Succinyladenosine | 0.742832538461538 | 416.2405 | 384.1133605 | Nucleosides, nucleotides, and analogues | POS |
| 338 | PC(22:6(4Z,7Z,10Z,13Z,16Z,19Z)/20:2(11Z,14Z)) | 0.741603 | 56.4945 | 858.5951173 | Lipids and lipid-like molecules | POS |
| 339 | PC(22:6(4Z,7Z,10Z,13Z,16Z,19Z)/20:4(5Z,8Z,11Z,14Z)) | 0.741414153846154 | 55.65155 | 855.5683951 | Lipids and lipid-like molecules | POS |
| 340 | PS(20:5(5Z,8Z,11Z,14Z,17Z)/18:1(9Z)) | 0.740363769230769 | 195.6145 | 807.6326261 | Lipids and lipid-like molecules | POS |
| 341 | (4S,5R,6R)-5-acetamido-2,4-dihydroxy-6-[(1R,2R)-1,2,3-trihydroxypropyl]oxane-2-carboxylic acid | 0.738178076923077 | 411.462 | 310.0848145 | Organic oxygen compounds | POS |
| 342 | LysoPC(20:1(11Z)) | 0.737167692307692 | 216.664 | 550.4907944 | Lipids and lipid-like molecules | POS |
| 343 | PI(20:3(8Z,11Z,14Z)/18:1(11Z)) | 0.732166538461538 | 210.546 | 887.5591136 | Lipids and lipid-like molecules | POS |
| 344 | trimethyl(2-oxoethyl)azanium | 0.730380230769231 | 414.171 | 102.0913196 | Organic nitrogen compounds | POS |
| 345 | PE(22:5(7Z,10Z,13Z,16Z,19Z)/22:5(7Z,10Z,13Z,16Z,19Z)) | 0.727943615384615 | 208.948 | 839.5589813 | Lipids and lipid-like molecules | POS |
| 346 | Isodesmosine | 0.727252 | 213.1955 | 525.3728196 | Organic acids and derivatives | POS |
| 347 | N-Acetyl-b-glucosaminylamine | 0.726491076923077 | 84.9222 | 221.1120372 | Organic oxygen compounds | POS |
| 348 | Eujambolin | 0.725429153846154 | 386.451 | 520.1863324 | Phenylpropanoids and polyketides | POS |
| 349 | Vinylacetylglycine | 0.725095076923077 | 438.444 | 143.0809935 | Organic acids and derivatives | POS |
| 350 | PC(P-18:1(11Z)/22:5(4Z,7Z,10Z,13Z,16Z)) | 0.724666153846154 | 149.6645 | 818.0697127 | Lipids and lipid-like molecules | POS |
| 351 | Diethylcarbamazine N-oxide | 0.716818769230769 | 382.82 | 216.0210759 | Organoheterocyclic compounds | POS |
| 352 | Pi-Methylimidazoleacetic acid | 0.713133307692308 | 350.321 | 140.8771506 | Organoheterocyclic compounds | POS |
| 353 | Azaspiracid 2 | 0.710334923076923 | 156.763 | 856.5327665 | Organoheterocyclic compounds | POS |
| 354 | LysoPI(18:0/0:0) | 0.709577384615385 | 265.886 | 601.3339681 | Lipids and lipid-like molecules | POS |
| 355 | 22-Acetylpriverogenin B | 0.708729538461538 | 52.9809 | 517.8538081 | Lipids and lipid-like molecules | POS |
| 356 | Valyl-Serine | 0.703945769230769 | 46.4227 | 204.1415543 | Organic acids and derivatives | POS |
| 357 | PS(16:0/20:1(11Z)) | 0.701560076923077 | 215.048 | 790.5551325 | Lipids and lipid-like molecules | POS |
| 358 | 5-L-Glutamyl-taurine | 0.700014384615385 | 381.917 | 254.9814725 | Organic acids and derivatives | POS |
| 359 | Serylhistidine | 0.699177692307692 | 358.555 | 243.1075566 | Organic acids and derivatives | POS |
| 360 | LysoPE(0:0/22:6(4Z,7Z,10Z,13Z,16Z,19Z)) | 0.692799923076923 | 213.199 | 526.3757711 | Lipids and lipid-like molecules | POS |
| 361 | hesperetin 3'-O-sulfate | 0.690269230769231 | 439.7715 | 382.886834 | Phenylpropanoids and polyketides | POS |
| 362 | Squamolone | 0.688045230769231 | 390.778 | 128.0703201 | Organoheterocyclic compounds | POS |
| 363 | PS(20:1(11Z)/20:1(11Z)) | 0.687971538461538 | 210.6195 | 844.6021072 | Lipids and lipid-like molecules | POS |
| 364 | 5-Aminopentanamide | 0.687439076923077 | 33.5398 | 117.0696206 | Organic acids and derivatives | POS |
| 365 | PS(18:0/18:4(6Z,9Z,12Z,15Z)) | 0.685078923076923 | 215.025 | 784.5086549 | Lipids and lipid-like molecules | POS |
| 366 | PC(P-16:0/22:6(4Z,7Z,10Z,13Z,16Z,19Z)) | 0.681212769230769 | 153.0475 | 789.6147909 | Lipids and lipid-like molecules | POS |
| 367 | 2-[(Methylthio)methyl]-2-butenal | 0.675211153846154 | 381.928 | 130.9572642 | Organic oxygen compounds | POS |
| 368 | PC(20:3(5Z,8Z,11Z)/16:0) | 0.670223846153846 | 152.558 | 784.580039 | Lipids and lipid-like molecules | POS |
| 369 | PC(14:0/14:0) | 0.670131538461538 | 68.9467 | 678.9506921 | Lipids and lipid-like molecules | POS |
| 370 | PS(22:4(7Z,10Z,13Z,16Z)/20:3(8Z,11Z,14Z)) | 0.669501384615385 | 212.484 | 862.5492923 | Lipids and lipid-like molecules | POS |
| 371 | CMP-2-aminoethylphosphonate | 0.668416461538461 | 423.716 | 430.2031696 | Organic oxygen compounds | POS |
| 372 | Leukotriene F4 | 0.667129076923077 | 452.7375 | 569.1398788 | Lipids and lipid-like molecules | POS |
| 373 | PS(18:0/16:1(9Z)) | 0.664812615384615 | 216.7785 | 762.5233083 | Lipids and lipid-like molecules | POS |
| 374 | Cyclovariegatin | 0.664703538461538 | 387.285 | 354.0754958 | Organoheterocyclic compounds | POS |
| 375 | PC(22:5(4Z,7Z,10Z,13Z,16Z)/22:1(13Z)) | 0.661131923076923 | 139.281 | 890.6589373 | Lipids and lipid-like molecules | POS |
| 376 | PE(P-16:0/20:5(5Z,8Z,11Z,14Z,17Z)) | 0.660796769230769 | 61.7537 | 722.5074146 | Lipids and lipid-like molecules | POS |
| 377 | dIMP | 0.653109384615385 | 436.138 | 332.0737193 | Nucleosides, nucleotides, and analogues | POS |
| 378 | 5-(dithiolan-3-yl)pentanamide | 0.651283153846154 | 152.481 | 206.1894613 | Organoheterocyclic compounds | POS |
| 379 | 2-Oxoarginine | 0.651185461538462 | 388.1095 | 174.0866194 | Organic acids and derivatives | POS |
| 380 | alpha-Tocopherolquinone | 0.651051692307692 | 30.9001 | 446.3975322 | Lipids and lipid-like molecules | POS |
| 381 | dCMP | 0.648729769230769 | 428.782 | 307.0773858 | Nucleosides, nucleotides, and analogues | POS |
| 382 | PC(22:4(7Z,10Z,13Z,16Z)/14:0) | 0.645120230769231 | 61.986 | 782.5666575 | Lipids and lipid-like molecules | POS |
| 383 | SM(d18:1/18:1(9Z)) | 0.643812923076923 | 161.7005 | 729.5216384 | Lipids and lipid-like molecules | POS |
| 384 | 1-Hexanethiol | 0.642414384615385 | 317.06 | 119.0893938 | Organosulfur compounds | POS |
| 385 | (2-butyl-1-benzofuran-3-yl)-[4-[2-(diethylamino)ethoxy]-3,5-diiodophenyl]methanone | 0.637548384615385 | 456.895 | 645.3292098 | Organic oxygen compounds | POS |
| 386 | PC(22:5(7Z,10Z,13Z,16Z,19Z)/16:0) | 0.633779153846154 | 146.3125 | 809.5837548 | Lipids and lipid-like molecules | POS |
| 387 | PE(18:0/22:6(4Z,7Z,10Z,13Z,16Z,19Z)) | 0.633300153846154 | 151.5525 | 792.5494036 | Lipids and lipid-like molecules | POS |
| 388 | SM(d18:0/18:1(9Z)) | 0.627189 | 168.396 | 731.9963281 | Lipids and lipid-like molecules | POS |
| 389 | (2S)-2-acetamido-3-(1H-imidazol-5-yl)propanoic acid | 0.624787538461538 | 22.0284 | 197.1260175 | Organic acids and derivatives | POS |
| 390 | PC(P-18:1(11Z)/14:0) | 0.623426384615385 | 64.5871 | 716.5550655 | Lipids and lipid-like molecules | POS |
| 391 | PC(22:6(4Z,7Z,10Z,13Z,16Z,19Z)/22:4(7Z,10Z,13Z,16Z)) | 0.622782 | 152.6275 | 882.5478691 | Lipids and lipid-like molecules | POS |
| 392 | PC(22:6(4Z,7Z,10Z,13Z,16Z,19Z)/20:0) | 0.621725692307692 | 142.774 | 862.627799 | Lipids and lipid-like molecules | POS |
| 393 | PC(20:4(8Z,11Z,14Z,17Z)/20:0) | 0.618818846153846 | 145.474 | 838.6265245 | Lipids and lipid-like molecules | POS |
| 394 | Moxonidine | 0.610237307692308 | 392.565 | 241.1534086 | Organoheterocyclic compounds | POS |
| 395 | PC(16:1(9Z)/16:0) | 0.610097846153846 | 199.9625 | 732.6067257 | Lipids and lipid-like molecules | POS |
| 396 | PC(18:1(11Z)/14:0) | 0.604933230769231 | 75.05835 | 732.5526368 | Lipids and lipid-like molecules | POS |
| 397 | PC(16:0/14:0) | 0.604448692307692 | 182.415 | 705.5802172 | Lipids and lipid-like molecules | POS |
| 398 | PC(16:1(9Z)/P-18:0) | 0.604304307692308 | 150.7045 | 744.5858003 | Lipids and lipid-like molecules | POS |
| 399 | Thymine | 0.600933615384615 | 311.7665 | 127.0248241 | Organoheterocyclic compounds | POS |
| 400 | Sulfasalazine | 0.600372769230769 | 347.293 | 398.7869017 | Organoheterocyclic compounds | POS |
| 401 | Theogallin | 0.599233846153846 | 439.857 | 344.9270496 | Organic oxygen compounds | POS |
| 402 | 2,5-Dihydro-2,4-dimethyloxazole | 0.595936384615385 | 91.2075 | 100.5263072 | Organoheterocyclic compounds | POS |
| 403 | PC(18:3(9Z,12Z,15Z)/18:0) | 0.589206769230769 | 196.616 | 784.6381771 | Lipids and lipid-like molecules | POS |
| 404 | 2-Methylpiperidine | 0.584829 | 268.7 | 100.09504 | Organoheterocyclic compounds | POS |
| 405 | PC(P-18:1(9Z)/18:3(6Z,9Z,12Z)) | 0.583864384615385 | 142.7745 | 766.5720146 | Lipids and lipid-like molecules | POS |
| 406 | PC(22:5(4Z,7Z,10Z,13Z,16Z)/16:0) | 0.583031307692308 | 59.7823 | 808.5821928 | Lipids and lipid-like molecules | POS |
| 407 | Garciduol A | 0.581375076923077 | 465.453 | 486.1144769 | Organoheterocyclic compounds | POS |
| 408 | PC(18:3(9Z,12Z,15Z)/18:1(11Z)) | 0.577014538461538 | 61.9917 | 783.5686449 | Lipids and lipid-like molecules | POS |
| 409 | PS(22:4(7Z,10Z,13Z,16Z)/15:0) | 0.574356615384615 | 62.7732 | 797.5833228 | Lipids and lipid-like molecules | POS |
| 410 | SM(d18:0/14:0) | 0.571910923076923 | 203.414 | 677.5474215 | Lipids and lipid-like molecules | POS |
| 411 | PE(P-16:0/16:1(9Z)) | 0.571220384615384 | 72.65905 | 674.5083495 | Lipids and lipid-like molecules | POS |
| 412 | Palmitoyl Serinol | 0.570801461538462 | 57.4953 | 330.2440549 | Lipids and lipid-like molecules | POS |
| 413 | PC(20:4(5Z,8Z,11Z,14Z)/P-18:1(11Z)) | 0.569657076923077 | 65.9491 | 793.0628767 | Lipids and lipid-like molecules | POS |
| 414 | 4-Aminohippuric acid | 0.565093230769231 | 331.408 | 184.0709085 | Benzenoids | POS |
| 415 | PC(22:5(7Z,10Z,13Z,16Z,19Z)/16:1(9Z)) | 0.564883384615385 | 60.2346 | 806.5674478 | Lipids and lipid-like molecules | POS |
| 416 | 1-O-Galloylglycerol | 0.564281230769231 | 451.642 | 244.1279658 | Benzenoids | POS |
| 417 | PS(16:0/18:1(11Z)) | 0.563901846153846 | 216.776 | 763.5288185 | Lipids and lipid-like molecules | POS |
| 418 | PC(20:5(5Z,8Z,11Z,14Z,17Z)/18:1(11Z)) | 0.563571538461539 | 147.9345 | 807.570686 | Lipids and lipid-like molecules | POS |
| 419 | PC(22:6(4Z,7Z,10Z,13Z,16Z,19Z)/22:2(13Z,16Z)) | 0.562652923076923 | 137.6335 | 886.6292885 | Lipids and lipid-like molecules | POS |
| 420 | PC(16:1(9Z)/16:1(9Z)) | 0.560267538461538 | 158.9235 | 730.5354502 | Lipids and lipid-like molecules | POS |
| 421 | PC(20:3(5Z,8Z,11Z)/20:4(5Z,8Z,11Z,14Z)) | 0.558834076923077 | 160.707 | 833.4938849 | Lipids and lipid-like molecules | POS |
| 422 | PC(22:5(4Z,7Z,10Z,13Z,16Z)/20:5(5Z,8Z,11Z,14Z,17Z)) | 0.558657769230769 | 145.441 | 854.6948276 | Lipids and lipid-like molecules | POS |
| 423 | SM(d18:1/14:0) | 0.558230846153846 | 203.48 | 675.5393779 | Lipids and lipid-like molecules | POS |
| 424 | N-[(4E,8E)-1,3-dihydroxyoctadeca-4,8-dien-2-yl]hexadecanamide | 0.555921692307692 | 32.7003 | 536.5038498 | Lipids and lipid-like molecules | POS |
| 425 | SM(d17:1/24:1(15Z)) | 0.555466692307692 | 195.5835 | 799.6664692 | Organic nitrogen compounds | POS |
| 426 | N-[(4E,8Z)-1,3-dihydroxyoctadeca-4,8-dien-2-yl]hexadecanamide 1-glucoside | 0.554917923076923 | 94.1468 | 698.5531785 | Lipids and lipid-like molecules | POS |
| 427 | PC(P-18:0/18:3(6Z,9Z,12Z)) | 0.553055538461539 | 152.8475 | 769.5542782 | Lipids and lipid-like molecules | POS |
| 428 | Androstenol | 0.552496307692308 | 65.5177 | 257.301813 | Lipids and lipid-like molecules | POS |
| 429 | PC(22:6(4Z,7Z,10Z,13Z,16Z,19Z)/P-16:0) | 0.550715692307692 | 64.3907 | 790.6177088 | Lipids and lipid-like molecules | POS |
| 430 | PC(18:3(6Z,9Z,12Z)/18:1(11Z)) | 0.548199846153846 | 149.8165 | 782.5667586 | Lipids and lipid-like molecules | POS |
| 431 | Dimethicone | 0.543667692307692 | 26.509 | 162.9721046 | Hydrocarbon derivatives | POS |
| 432 | lysoPC(26:1(5Z)) | 0.541610846153846 | 231.739 | 635.133971 | Lipids and lipid-like molecules | POS |
| 433 | PC(22:4(7Z,10Z,13Z,16Z)/22:1(13Z)) | 0.541152384615385 | 138.302 | 892.6742133 | Lipids and lipid-like molecules | POS |
| 434 | gamma-Glutamylphenylalanine | 0.537410769230769 | 358.592 | 295.1270903 | Organic acids and derivatives | POS |
| 435 | 3,4-Dihydroxy-2-hydroxymethyl-1-pyrrolidinepropanamide | 0.532206615384615 | 91.2486 | 205.0389286 | Organoheterocyclic compounds | POS |
| 436 | 5-Aminoimidazole ribonucleotide | 0.532147846153846 | 404.604 | 296.0643333 | Organic oxygen compounds | POS |
| 437 | PC(22:5(7Z,10Z,13Z,16Z,19Z)/18:1(11Z)) | 0.531012230769231 | 58.3275 | 835.5997173 | Lipids and lipid-like molecules | POS |
| 438 | PC(22:5(7Z,10Z,13Z,16Z,19Z)/14:0) | 0.520027923076923 | 143.129 | 779.574134 | Lipids and lipid-like molecules | POS |
| 439 | Cochliophilin A | 0.517656 | 351.202 | 282.8378386 | Phenylpropanoids and polyketides | POS |
| 440 | Pteroside B | 0.515830538461538 | 138.3325 | 381.1414934 | Organic oxygen compounds | POS |
| 441 | N-(4-aminobutyl)acetamide | 0.510730769230769 | 319.736 | 131.1003637 | Organic acids and derivatives | POS |
| 442 | PE(16:0/14:0) | 0.507212 | 166.593 | 664.4883987 | Lipids and lipid-like molecules | POS |
| 443 | (2R,3S)-5-[3-(tert-butylamino)-2-hydroxypropoxy]-1,2,3,4-tetrahydronaphthalene-2,3-diol | 0.504864846153846 | 247.102 | 309.2156136 | Benzenoids | POS |
| 444 | PC(22:4(7Z,10Z,13Z,16Z)/22:5(4Z,7Z,10Z,13Z,16Z)) | 0.498438615384615 | 64.4687 | 884.5639046 | Lipids and lipid-like molecules | POS |
| 445 | (3beta,5alpha,6beta,22E,24R)-23-Methylergosta-7,22-diene-3,5,6-triol | 0.498345615384615 | 30.8879 | 445.3664175 | Lipids and lipid-like molecules | POS |
| 446 | 2-amino-5-(ethylamino)-5-oxopentanoic acid | 0.494760384615385 | 48.3094 | 175.0858507 | Organic acids and derivatives | POS |
| 447 | Vignatic acid A | 0.487441923076923 | 169.711 | 554.0551898 | Organic acids and derivatives | POS |
| 448 | Chrysoeriol 7-O-(6''-malonyl-glucoside) | 0.487304384615385 | 381.954 | 548.8645214 | Phenylpropanoids and polyketides | POS |
| 449 | PC(20:4(8Z,11Z,14Z,17Z)/18:1(11Z)) | 0.470923153846154 | 59.7542 | 809.5839188 | Lipids and lipid-like molecules | POS |
| 450 | PC(24:1(15Z)/18:4(6Z,9Z,12Z,15Z)) | 0.469752846153846 | 57.17925 | 864.6417632 | Lipids and lipid-like molecules | POS |
| 451 | Paraoxon | 0.466675153846154 | 334.153 | 275.1699208 | Benzenoids | POS |
| 452 | Dodecanoylcarnitine | 0.461122153846154 | 186.8105 | 343.2938959 | Lipids and lipid-like molecules | POS |
| 453 | LysoPC(24:1(15Z)) | 0.458961230769231 | 214.083 | 605.5488336 | Lipids and lipid-like molecules | POS |
| 454 | Alendronic acid | 0.442771846153846 | 244.2985 | 250.0233687 | Organic acids and derivatives | POS |
| 455 | PC(20:4(8Z,11Z,14Z,17Z)/20:3(8Z,11Z,14Z)) | 0.438300230769231 | 58.4028 | 833.5861857 | Lipids and lipid-like molecules | POS |
| 456 | Valyl-Methionine | 0.431237 | 217.578 | 248.14625 | Organic acids and derivatives | POS |
| 457 | Thiomorpholine 3-carboxylate | 0.418067 | 316.172 | 148.0961225 | Organic acids and derivatives | POS |
| 458 | (4R,4aR,7S,7aR,12bS)-3-methyl-2,4,4a,7,7a,13-hexahydro-1H-4,12-methanobenzofuro[3,2-e]isoquinoline-7,9-diol | 0.398814384615385 | 52.2807 | 285.1787848 | Alkaloids and derivatives | POS |
| 459 | CDP-glycerol | 0.397090461538462 | 178.043 | 478.0648569 | Lipids and lipid-like molecules | POS |
| 460 | Tricrocin | 0.392466076923077 | 346.803 | 814.5665026 | Lipids and lipid-like molecules | POS |
| 461 | Ethyl oleate | 0.999971846153846 | 58.4691 | 310.1734259 | Lipids and lipid-like molecules | NEG |
| 462 | Malonoben | 0.999971538461538 | 37.6319 | 280.2353233 | Benzenoids | NEG |
| 463 | (Z)-octadec-9-enoic acid | 0.999765846153846 | 38.017 | 281.247649 | Lipids and lipid-like molecules | NEG |
| 464 | (4S)-2,6-dioxo-1,3-diazinane-4-carboxylic acid | 0.999631615384615 | 347.197 | 156.4589742 | Organic acids and derivatives | NEG |
| 465 | Hypogeic acid | 0.999569153846154 | 47.1693 | 253.2166245 | Lipids and lipid-like molecules | NEG |
| 466 | 3-isothiocyanatoprop-1-ene | 0.999549846153846 | 188.173 | 98.02372379 | Organosulfur compounds | NEG |
| 467 | 2-oxopropanoic acid | 0.999535846153846 | 121.039 | 86.02382937 | Organic acids and derivatives | NEG |
| 468 | 15-Methylpalmitate | 0.999518615384615 | 44.5613 | 268.9961564 | Organic compounds | NEG |
| 469 | 2,6-diaminohexanoic acid | 0.999416769230769 | 540.894 | 145.0971394 | Organic acids and derivatives | NEG |
| 470 | Ricinoleic acid | 0.999406384615385 | 50.00705 | 297.2428288 | Lipids and lipid-like molecules | NEG |
| 471 | Adrenic acid | 0.999333461538461 | 37.6189 | 331.2630598 | Lipids and lipid-like molecules | NEG |
| 472 | (Z)-hexadec-9-enoic acid | 0.999204615384615 | 36.5216 | 253.216576 | Lipids and lipid-like molecules | NEG |
| 473 | 1H-pyrimidine-2,4-dione | 0.999146538461539 | 75.6004 | 111.018901 | Organoheterocyclic compounds | NEG |
| 474 | Arachidonic acid | 0.999039076923077 | 37.538 | 303.2319022 | Lipids and lipid-like molecules | NEG |
| 475 | Hypotaurine | 0.998702923076923 | 388.4905 | 107.0240562 | Organic acids and derivatives | NEG |
| 476 | ethyl butanoate | 0.998672307692308 | 73.3587 | 115.0753815 | Lipids and lipid-like molecules | NEG |
| 477 | 4-dodecylbenzenesulfonic acid | 0.998575538461538 | 26.8475 | 325.1835757 | Benzenoids | NEG |
| 478 | 2-aminoethyl dihydrogen phosphate | 0.998369538461538 | 494.381 | 140.0108859 | Organic acids and derivatives | NEG |
| 479 | pentanoic acid | 0.998068 | 91.6053 | 101.0597962 | Lipids and lipid-like molecules | NEG |
| 480 | Leucinic acid | 0.996974692307692 | 120.084 | 131.0703676 | Lipids and lipid-like molecules | NEG |
| 481 | 16-Hydroxy hexadecanoic acid | 0.996831307692308 | 26.8286 | 271.8430855 | Lipids and lipid-like molecules | NEG |
| 482 | p-Cresol | 0.996105 | 210.9735 | 107.0492352 | Benzenoids | NEG |
| 483 | 7,9-dihydro-3H-purine-2,6,8-trione | 0.995941076923077 | 368.542 | 166.0215952 | Organoheterocyclic compounds | NEG |
| 484 | Docosahexaenoic acid | 0.995745307692308 | 57.0071 | 328.2305939 | Lipids and lipid-like molecules | NEG |
| 485 | pentadecanoic acid | 0.995466307692308 | 47.8856 | 241.21647 | Lipids and lipid-like molecules | NEG |
| 486 | Dehydroepiandrosterone sulfate | 0.995219 | 45.5574 | 366.2699534 | Lipids and lipid-like molecules | NEG |
| 487 | Sulfaphenazole | 0.995148692307692 | 58.6576 | 312.2254022 | Organoheterocyclic compounds | NEG |
| 488 | Hydrocinnamic acid | 0.994963153846154 | 88.3952 | 149.0597874 | Phenylpropanoids and polyketides | NEG |
| 489 | 2-hydroxybutanoic acid | 0.994913307692308 | 186.2825 | 103.0342277 | Organic acids and derivatives | NEG |
| 490 | tetradecanoic acid | 0.994720153846154 | 48.0603 | 227.2008215 | Lipids and lipid-like molecules | NEG |
| 491 | cis-Vaccenic acid | 0.994505307692308 | 129.862 | 281.2476311 | Lipids and lipid-like molecules | NEG |
| 492 | benzoic acid | 0.994420692307692 | 36.52815 | 121.0285001 | Benzenoids | NEG |
| 493 | (Z)-but-2-enedioic acid | 0.994260461538461 | 51.5669 | 114.0186875 | Organic acids and derivatives | NEG |
| 494 | 2-amino-2-methylpropanoic acid | 0.993705384615385 | 382.272 | 101.9798111 | Organic acids and derivatives | NEG |
| 495 | D-Tryptophan | 0.992893307692308 | 284.2405 | 203.022188 | Organoheterocyclic compounds | NEG |
| 496 | 4-Hydroxyproline | 0.992862307692308 | 361.2105 | 130.0499836 | Organic acids and derivatives | NEG |
| 497 | Guanosine | 0.991809923076923 | 280.6225 | 282.0835093 | Nucleosides, nucleotides, and analogues | NEG |
| 498 | 2,3-dihydroxypropanal | 0.991214461538461 | 239.378 | 88.79651591 | Organic oxygen compounds | NEG |
| 499 | tridecanoic acid | 0.991079076923077 | 21.5088 | 213.0141721 | Lipids and lipid-like molecules | NEG |
| 500 | 3-methyl-2-oxobutanoic acid | 0.990969153846154 | 94.5523 | 115.0390869 | Organic acids and derivatives | NEG |
| 501 | Prostaglandin D2 | 0.990445769230769 | 27.7679 | 351.1838948 | Lipids and lipid-like molecules | NEG |
| 502 | nonanoic acid | 0.990189307692308 | 52.515 | 157.1224501 | Lipids and lipid-like molecules | NEG |
| 503 | 4-Hydroxy tolbutamide | 0.989718307692308 | 277.782 | 285.0928048 | Benzenoids | NEG |
| 504 | (E)-prop-1-ene-1,2,3-tricarboxylic acid | 0.988754384615384 | 90.75485 | 173.0082477 | Organic acids and derivatives | NEG |
| 505 | phosphoric acid | 0.988194153846154 | 438.2055 | 96.9590191 | Homogeneous non-metal compounds | NEG |
| 506 | Glutathione | 0.987961 | 382.2635 | 306.0252978 | Organic acids and derivatives | NEG |
| 507 | 15-Keto-13,14-dihydroprostaglandin A2 | 0.987573461538461 | 53.87035 | 332.7270687 | Lipids and lipid-like molecules | NEG |
| 508 | 13-OxoODE | 0.987422461538461 | 50.6935 | 293.2111984 | Lipids and lipid-like molecules | NEG |
| 509 | Orotidine | 0.987368153846154 | 354.3725 | 286.1874444 | Nucleosides, nucleotides, and analogues | NEG |
| 510 | Indoleacetaldehyde | 0.987017923076923 | 5.0604 | 157.9881722 | Organoheterocyclic compounds | NEG |
| 511 | Taurocholic acid | 0.987004923076923 | 216.9605 | 514.2833788 | Lipids and lipid-like molecules | NEG |
| 512 | hexadecanoic acid | 0.986905153846154 | 347.197 | 254.9668982 | Lipids and lipid-like molecules | NEG |
| 513 | Ethyl tetradecanoate | 0.986500615384615 | 108.5215 | 255.0111868 | Lipids and lipid-like molecules | NEG |
| 514 | [(2R,3S,4R)-3,4,5-trihydroxyoxolan-2-yl]methyl dihydrogen phosphate | 0.985122846153846 | 438.356 | 229.0820952 | Organic oxygen compounds | NEG |
| 515 | Cytidine 2',3'-cyclic phosphate | 0.984752769230769 | 329.954 | 303.0755814 | Nucleosides, nucleotides, and analogues | NEG |
| 516 | Myristoleic acid | 0.984706923076923 | 34.79495 | 224.5626596 | Lipids and lipid-like molecules | NEG |
| 517 | Fructose-1P | 0.983674692307692 | 408.4675 | 179.0551288 | Organooxygen compounds | NEG |
| 518 | Dehydroascorbic acid | 0.983629923076923 | 162.498 | 172.0202027 | Organoheterocyclic compounds | NEG |
| 519 | 1H-indol-3-yl hydrogen sulfate | 0.983574538461538 | 41.8997 | 211.0350704 | Organic acids and derivatives | NEG |
| 520 | 5Z-Dodecenoic acid | 0.98331 | 71.01245 | 198.0818984 | Lipids and lipid-like molecules | NEG |
| 521 | N-Acetylaspartylglutamic acid | 0.982998615384615 | 458.7565 | 302.1462182 | Organic acids and derivatives | NEG |
| 522 | 2-Ketohexanoic acid | 0.982885923076923 | 36.60255 | 129.0547224 | Organic acids and derivatives | NEG |
| 523 | 2-methylidenebutanedioic acid | 0.979073615384615 | 64.74515 | 129.0182813 | Lipids and lipid-like molecules | NEG |
| 524 | Methylsuccinic acid | 0.977761615384615 | 361.7115 | 131.0339263 | Lipids and lipid-like molecules | NEG |
| 525 | Phosphocreatine | 0.977240769230769 | 452.286 | 210.0275299 | Organic acids and derivatives | NEG |
| 526 | Sucrose | 0.976514153846154 | 407.417 | 341.1082944 | Organic oxygen compounds | NEG |
| 527 | Indoleacrylic acid | 0.975351230769231 | 239.307 | 186.0446391 | Organoheterocyclic compounds | NEG |
| 528 | 2-Ketobutyric acid | 0.974867769230769 | 80.72245 | 101.0233527 | Organic acids and derivatives | NEG |
| 529 | (10E,12Z)-(9S)-9-Hydroperoxyoctadeca-10,12-dienoic acid | 0.974092307692308 | 58.8604 | 311.2217868 | Lipids and lipid-like molecules | NEG |
| 530 | Deoxycholic acid | 0.972914538461539 | 101.8075 | 391.072699 | Lipids and lipid-like molecules | NEG |
| 531 | Inosinic acid | 0.972742615384615 | 460.6475 | 347.0390598 | Nucleosides, nucleotides, and analogues | NEG |
| 532 | Prostaglandin B2 | 0.972395461538462 | 71.0203 | 333.2062769 | Lipids and lipid-like molecules | NEG |
| 533 | 2-[[(4R)-4-[(3R,5R,8R,9S,10S,12S,13R,14S,17R)-3,12-dihydroxy-10,13-dimethyl-2,3,4,5,6,7,8,9,11,12,14,15,16,17-tetradecahydro-1H-cyclopenta[a]phenanthren-17-yl]pentanoyl]amino]ethanesulfonic acid | 0.972217384615384 | 169.626 | 498.3240743 | Lipids and lipid-like molecules | NEG |
| 534 | Ethyl Arachidonate | 0.969555923076923 | 33.0233 | 332.172896 | Lipids and lipid-like molecules | NEG |
| 535 | 4-hydroxybenzaldehyde | 0.969037538461538 | 57.8353 | 121.0396913 | Organic oxygen compounds | NEG |
| 536 | Oxoglutaric acid | 0.967707076923077 | 391.114 | 144.0268034 | Organic acids and derivatives | NEG |
| 537 | D-Xylulose | 0.967602076923077 | 274.451 | 148.0473594 | Organooxygen compounds | NEG |
| 538 | 2-acetamidopentanedioic acid | 0.966882461538461 | 397.476 | 188.0554521 | Organic acids and derivatives | NEG |
| 539 | Nelarabine | 0.962810461538462 | 217.5355 | 295.2270369 | Nucleosides, nucleotides, and analogues | NEG |
| 540 | L-3-Phenyllactic acid | 0.962727 | 95.21185 | 165.0547749 | Phenylpropanoids and polyketides | NEG |
| 541 | 2-hydroxy-3-(4-hydroxyphenyl)propanoic acid | 0.960912153846154 | 174.231 | 180.9993626 | Phenylpropanoids and polyketides | NEG |
| 542 | 3-hydroxy-3-methylpentanedioic acid | 0.958627769230769 | 425.598 | 161.0445597 | Lipids and lipid-like molecules | NEG |
| 543 | L-Malic acid | 0.958288615384615 | 438.495 | 133.0132835 | Organic acids and derivatives | NEG |
| 544 | 2-amino-1,7-dihydropurin-6-one | 0.958007846153846 | 244.667 | 150.0410176 | Organoheterocyclic compounds | NEG |
| 545 | (2S)-2-amino-3-phenylpropanoic acid | 0.954848384615385 | 274.6785 | 164.0706151 | Organic acids and derivatives | NEG |
| 546 | Galactose 1-phosphate | 0.948832 | 499.631 | 259.0214556 | Organic oxygen compounds | NEG |
| 547 | (3R,4R)-3,4-dihydroxyoxolan-2-one | 0.946592538461539 | 397.4995 | 117.0182547 | Organoheterocyclic compounds | NEG |
| 548 | (3S,4S,5S,6R)-6-(hydroxymethyl)oxane-2,3,4,5-tetrol | 0.945164076923077 | 347.199 | 179.0369888 | Organic oxygen compounds | NEG |
| 549 | D-Erythrose 4-phosphate | 0.944851307692308 | 424.5225 | 198.0162768 | Organic oxygen compounds | NEG |
| 550 | Creatinine | 0.944823076923077 | 366.132 | 112.0506548 | Organic acids and derivatives | NEG |
| 551 | 6-Keto-prostaglandin F1a | 0.944711 | 211.345 | 369.154293 | Lipids and lipid-like molecules | NEG |
| 552 | 4-hydroxy-3-methoxybenzaldehyde | 0.944406538461538 | 75.9489 | 151.0114772 | Benzenoids | NEG |
| 553 | 16(17)-EpDPE | 0.943707 | 56.19715 | 342.2460752 | Lipids and lipid-like molecules | NEG |
| 554 | 2-(3,4-dihydroxy-5-methoxyphenyl)-3,5,7-trihydroxychromen-4-one | 0.943032461538462 | 434.7115 | 330.0599037 | Phenylpropanoids and polyketides | NEG |
| 555 | (2S,3S,4S,5R,6R)-6-[[[(2S,3R,4S,5S)-5-(2,4-dioxopyrimidin-1-yl)-3,4-dihydroxyoxolan-2-yl]methoxy-hydroxyphosphoryl]oxy-hydroxyphosphoryl]oxy-3,4,5-trihydroxyoxane-2-carboxylic acid | 0.942484384615385 | 485.9005 | 579.0269978 | Nucleosides, nucleotides, and analogues | NEG |
| 556 | 3-Carboxy-4-methyl-5-propyl-2-furanpropionic acid | 0.939974 | 182.756 | 238.0941489 | Lipids and lipid-like molecules | NEG |
| 557 | 2,3-dihydroxypropanoic acid | 0.938789846153846 | 318.264 | 105.0183301 | Organic oxygen compounds | NEG |
| 558 | [(2R,3R,4S,5R,6R)-3,4,5-trihydroxy-6-(hydroxymethyl)oxan-2-yl] dihydrogen phosphate | 0.938002307692308 | 469.52 | 259.021435 | Organic oxygen compounds | NEG |
| 559 | Uridine diphosphate-N-acetylglucosamine | 0.937807461538462 | 440.384 | 606.0748985 | Nucleosides, nucleotides, and analogues | NEG |
| 560 | butanedioic acid | 0.936085076923077 | 90.3022 | 118.0135914 | Organic acids and derivatives | NEG |
| 561 | 5-[(2S,3R,4S,5R)-3,4-dihydroxy-5-(hydroxymethyl)oxolan-2-yl]-1H-pyrimidine-2,4-dione | 0.931063692307692 | 228.642 | 243.0171011 | Nucleosides, nucleotides, and analogues | NEG |
| 562 | (2S)-2-amino-3-hydroxypropanoic acid | 0.929898307692308 | 407.2235 | 104.0342505 | Organic acids and derivatives | NEG |
| 563 | L-Gulonolactone | 0.928000461538461 | 132.605 | 177.0216243 | Organoheterocyclic compounds | NEG |
| 564 | 1,1,2,2,3,3,4,4,5,5,6,6,7,7,8,8,8-heptadecafluorooctane-1-sulfonic acid | 0.926752615384615 | 22.40235 | 498.9296667 | Organohalogen compounds | NEG |
| 565 | Succinic acid semialdehyde | 0.924528384615384 | 274.428 | 101.0233313 | Lipids and lipid-like molecules | NEG |
| 566 | [(2S,3R,4S,5S,6R)-3,4,5-trihydroxy-6-(hydroxymethyl)oxan-2-yl] (1E)-3-phenyl-N-sulfooxypropanimidothioate | 0.923857384615385 | 188.428 | 459.0301566 | Organooxygen compounds | NEG |
| 567 | Cholesterol sulfate | 0.923014 | 25.05375 | 465.3027192 | Lipids and lipid-like molecules | NEG |
| 568 | Malonic acid | 0.919226153846154 | 401.049 | 103.0026055 | Organic acids and derivatives | NEG |
| 569 | 2-Methylguanosine | 0.918122769230769 | 245.605 | 296.0992151 | Nucleosides, nucleotides, and analogues | NEG |
| 570 | Butabarbital | 0.913110230769231 | 171.399 | 210.8414659 | Organoheterocyclic compounds | NEG |
| 571 | 2-hydroxypropane-1,2,3-tricarboxylic acid | 0.910235461538462 | 90.351 | 190.0348597 | Organic acids and derivatives | NEG |
| 572 | 12-Methyltridecanoic acid | 0.909683615384615 | 32.15005 | 227.0139737 | Lipids and lipid-like molecules | NEG |
| 573 | 9,10-DHOME | 0.907163846153846 | 64.8659 | 313.2377209 | Lipids and lipid-like molecules | NEG |
| 574 | [(2R,3S,4R,5R)-5-(6-aminopurin-9-yl)-3,4-dihydroxyoxolan-2-yl]methyl phosphono hydrogen phosphate | 0.906090615384615 | 486.676 | 426.0213819 | Nucleosides, nucleotides, and analogues | NEG |
| 575 | N-Acetyl-glucosamine 1-phosphate | 0.905786846153846 | 463.12 | 300.0480686 | Organic oxygen compounds | NEG |
| 576 | Saccharin | 0.896979538461538 | 5.06346 | 181.008557 | Organoheterocyclic compounds | NEG |
| 577 | [(2R,3S,4R,5R)-5-(6-aminopurin-9-yl)-4-hydroxy-2-(hydroxymethyl)oxolan-3-yl] dihydrogen phosphate | 0.895842461538461 | 401.224 | 345.2131827 | Nucleosides, nucleotides, and analogues | NEG |
| 578 | Nicotinic acid mononucleotide | 0.892791384615385 | 175.773 | 334.0729119 | Organic oxygen compounds | NEG |
| 579 | [(2R,3S,4R,5R)-5-(6-aminopurin-9-yl)-3,4-dihydroxyoxolan-2-yl]methyl dihydrogen phosphate | 0.891254846153846 | 477.348 | 345.5783434 | Nucleosides, nucleotides, and analogues | NEG |
| 580 | Maltotriose | 0.889974384615384 | 467.1875 | 503.1617101 | Organic oxygen compounds | NEG |
| 581 | 1,7-dimethyl-3H-purine-2,6-dione | 0.888621076923077 | 94.2867 | 179.0337771 | Organoheterocyclic compounds | NEG |
| 582 | 2-aminopentanedioic acid | 0.888310692307692 | 442.209 | 146.0448432 | Organic acids and derivatives | NEG |
| 583 | [(2R,3S,4R,5R)-3,4-dihydroxy-5-(6-oxo-1H-purin-9-yl)oxolan-2-yl]methyl dihydrogen phosphate | 0.884071538461538 | 427.2645 | 347.004335 | Nucleosides, nucleotides, and analogues | NEG |
| 584 | Cefuroxime | 0.883591230769231 | 180.939 | 423.0612563 | Organoheterocyclic compounds | NEG |
| 585 | 3-(3-Hydroxyphenyl)propanoic acid | 0.881096923076923 | 18.7786 | 164.0818903 | Phenylpropanoids and polyketides | NEG |
| 586 | 2-butan-2-yl-4,6-dinitrophenol | 0.880384 | 155.506 | 238.0712129 | Benzenoids | NEG |
| 587 | [(2R,3S,4R,5R)-5-(4-amino-2-oxopyrimidin-1-yl)-3,4-dihydroxyoxolan-2-yl]methyl dihydrogen phosphate | 0.877420076923077 | 488.705 | 321.0646517 | Nucleosides, nucleotides, and analogues | NEG |
| 588 | (2S)-2-hydroxybutanedioic acid | 0.876087846153846 | 495.145 | 132.0291913 | Organic acids and derivatives | NEG |
| 589 | (R)-3-Hydroxybutyric acid | 0.874973923076923 | 430.469 | 103.0390554 | Organic acids and derivatives | NEG |
| 590 | 2-(5-hydroxy-1H-indol-3-yl)acetic acid | 0.870321153846154 | 284.42 | 189.9787474 | Organoheterocyclic compounds | NEG |
| 591 | Azelaic acid | 0.868742384615384 | 347.196 | 187.0003785 | Lipids and lipid-like molecules | NEG |
| 592 | 3-methyl-2-oxopentanoic acid | 0.867043692307692 | 53.208 | 129.0546204 | Organic acids and derivatives | NEG |
| 593 | Deoxyguanosine | 0.863120153846154 | 227.86 | 265.1184602 | Nucleosides, nucleotides, and analogues | NEG |
| 594 | Threonic acid | 0.862469538461538 | 342.238 | 135.0288316 | Organic oxygen compounds | NEG |
| 595 | Imidazoleacetic acid | 0.860749461538461 | 75.9659 | 124.9704215 | Organoheterocyclic compounds | NEG |
| 596 | 3-amino-2-methylpropanoic acid | 0.851314384615385 | 363.517 | 102.018567 | Organic acids and derivatives | NEG |
| 597 | (13E)-11a-Hydroxy-9,15-dioxoprost-13-enoic acid | 0.850659538461538 | 71.1774 | 351.216948 | Lipids and lipid-like molecules | NEG |
| 598 | 2-(1H-indol-3-yl)acetaldehyde | 0.846633769230769 | 56.03795 | 158.2008604 | Organoheterocyclic compounds | NEG |
| 599 | 2-propylpentanoic acid | 0.839032 | 54.3759 | 142.9975848 | Lipids and lipid-like molecules | NEG |
| 600 | D-Maltose | 0.829785923076923 | 417.216 | 341.1456951 | Organic oxygen compounds | NEG |
| 601 | N-Acetyl-a-neuraminic acid | 0.828046384615384 | 411.7845 | 308.0712529 | Organic oxygen compounds | NEG |
| 602 | Indole-3-propionic acid | 0.827957230769231 | 49.9037 | 188.0192392 | Organoheterocyclic compounds | NEG |
| 603 | (E)-9,10-dihydroxyoctadec-12-enoic acid | 0.824667 | 88.11225 | 313.0658574 | Lipids and lipid-like molecules | NEG |
| 604 | D-Glycero-D-galacto-heptitol | 0.824576769230769 | 366.1395 | 212.0646161 | Organic oxygen compounds | NEG |
| 605 | Thymidine | 0.823100153846154 | 117.482 | 241.0689923 | Nucleosides, nucleotides, and analogues | NEG |
| 606 | (E)-3-(4-hydroxyphenyl)prop-2-enoic acid | 0.810774384615385 | 53.4183 | 162.9188944 | Phenylpropanoids and polyketides | NEG |
| 607 | 1H-indole-2,3-dione | 0.805483076923077 | 84.4598 | 146.964976 | Organoheterocyclic compounds | NEG |
| 608 | 2-[(2-aminoacetyl)amino]-3-methylpentanoic acid | 0.804805923076923 | 301.3105 | 187.1079789 | Organic acids and derivatives | NEG |
| 609 | 2-hydroxy-2-methylbutanedioic acid | 0.802047153846154 | 106.9115 | 147.0288571 | Lipids and lipid-like molecules | NEG |
| 610 | [2-[(2-amino-6-oxo-1H-purin-9-yl)methoxy]-3-hydroxypropyl] (2S)-2-amino-3-methylbutanoate | 0.801998153846154 | 205.6005 | 352.9440847 | Organic acids and derivatives | NEG |
| 611 | Theaflavin | 0.799824 | 422.829 | 563.032306 | Phenylpropanoids and polyketides | NEG |
| 612 | [(2R,3S,5R)-5-(2,4-dioxopyrimidin-1-yl)-3-hydroxyoxolan-2-yl]methyl dihydrogen phosphate | 0.792922230769231 | 238.507 | 307.0334973 | Nucleosides, nucleotides, and analogues | NEG |
| 613 | Phenylalanylproline | 0.781669692307692 | 246.556 | 261.028243 | Organic acids and derivatives | NEG |
| 614 | Gamma-Linolenic acid | 0.781501384615384 | 58.9127 | 278.1251761 | Lipids and lipid-like molecules | NEG |
| 615 | (5R)-5-[(1S)-1,2-dihydroxyethyl]oxolane-2,3,4-trione | 0.778328769230769 | 107.8415 | 172.0242471 | Organoheterocyclic compounds | NEG |
| 616 | Cytidine | 0.777182307692308 | 371.53 | 242.1136786 | Nucleosides, nucleotides, and analogues | NEG |
| 617 | 9-[(2R,3R,4S,5R)-3,4-dihydroxy-5-(hydroxymethyl)oxolan-2-yl]-2-(dimethylamino)-1H-purin-6-one | 0.772072384615385 | 174.936 | 309.1699169 | Nucleosides, nucleotides, and analogues | NEG |
| 618 | 4-amino-1-[(2R,3R,4S,5R)-3,4-dihydroxy-5-(hydroxymethyl)oxolan-2-yl]pyrimidin-2-one | 0.771361230769231 | 430.996 | 242.0064381 | Nucleosides, nucleotides, and analogues | NEG |
| 619 | (2S)-5-oxopyrrolidine-2-carboxylic acid | 0.768432461538461 | 353.4535 | 128.0343116 | Organic acids and derivatives | NEG |
| 620 | Uridine diphosphategalactose | 0.767324 | 430.199 | 564.1070703 | Nucleosides, nucleotides, and analogues | NEG |
| 621 | 2-Keto-3-deoxy-D-gluconic acid | 0.75841 | 179.271 | 176.9799663 | Organic acids and derivatives | NEG |
| 622 | (2S)-2-acetamidopropanoic acid | 0.754681307692308 | 403.7635 | 130.0499804 | Organic acids and derivatives | NEG |
| 623 | D-Alanyl-D-alanine | 0.745085692307692 | 323.728 | 158.1175978 | Organic acids and derivatives | NEG |
| 624 | Nitrofurazone | 0.742195846153846 | 160.859 | 196.8418585 | Organoheterocyclic compounds | NEG |
| 625 | Cytidine monophosphate N-acetylneuraminic acid | 0.741352923076923 | 453.167 | 613.1398362 | Nucleosides, nucleotides, and analogues | NEG |
| 626 | [(2R,3S,4S,5R)-3,4,5,6-tetrahydroxyoxan-2-yl]methyl dihydrogen phosphate | 0.740940769230769 | 70.3464 | 258.144985 | Organic oxygen compounds | NEG |
| 627 | 2-hydroxy-2-methylpropanoic acid | 0.739202153846154 | 285.1845 | 103.0390352 | Organic acids and derivatives | NEG |
| 628 | L-2-Hydroxyglutaric acid | 0.737296076923077 | 376.907 | 146.9649435 | Lipids and lipid-like molecules | NEG |
| 629 | Quinolinic acid | 0.736701846153846 | 401.313 | 166.0135286 | Organoheterocyclic compounds | NEG |
| 630 | (2R,3R,4S,5S,6R)-2-[(2S,3R,4S,5S,6S)-4,5-dihydroxy-2-(hydroxymethyl)-6-[(2S,3R,4S,5S)-4,5,6-trihydroxy-2-(hydroxymethyl)oxan-3-yl]oxyoxan-3-yl]oxy-6-(hydroxymethyl)oxane-3,4,5-triol | 0.728707769230769 | 439.626 | 504.1232239 | Organic oxygen compounds | NEG |
| 631 | (10E,12E)-9-oxooctadeca-10,12-dienoic acid | 0.725796923076923 | 93.7833 | 292.8907179 | Lipids and lipid-like molecules | NEG |
| 632 | Clavulanate | 0.716788307692308 | 30.34795 | 198.0056967 | Organic acids and derivatives | NEG |
| 633 | Buprenorphine | 0.716533 | 208.686 | 465.3170064 | Benzenoids | NEG |
| 634 | 11-Dehydro-thromboxane B2 | 0.716374230769231 | 106.7055 | 367.211438 | Lipids and lipid-like molecules | NEG |
| 635 | 2',4',6'-Trihydroxyacetophenone | 0.714215692307692 | 49.89995 | 167.0340208 | Benzenoids | NEG |
| 636 | Citric acid | 0.713356230769231 | 174.912 | 191.0187036 | Organic acids and derivatives | NEG |
| 637 | 2-(3-methylbutanoylamino)acetic acid | 0.713178846153846 | 200.683 | 158.0812815 | Organic acids and derivatives | NEG |
| 638 | 2-acetamido-4-methylsulfanylbutanoic acid | 0.703158615384615 | 205.597 | 189.0952091 | Organic acids and derivatives | NEG |
| 639 | Bovinic acid | 0.698626307692308 | 659.909 | 278.9963108 | Lipids and lipid-like molecules | NEG |
| 640 | 3-methylbutanoic acid | 0.694907153846154 | 3.266495 | 101.9798606 | Lipids and lipid-like molecules | NEG |
| 641 | Gluconolactone | 0.683925769230769 | 46.82155 | 177.0393987 | Organic oxygen compounds | NEG |
| 642 | LysoPA(16:0/0:0) | 0.669920076923077 | 189.791 | 409.067157 | Lipids and lipid-like molecules | NEG |
| 643 | Methylgingerol | 0.667779230769231 | 37.5183 | 306.2511099 | Benzenoids | NEG |
| 644 | Hydroxypyruvic acid | 0.665757 | 90.5316 | 103.0026054 | Organic acids and derivatives | NEG |
| 645 | 4-methyl-2-oxopentanoic acid | 0.663856230769231 | 126.0185 | 130.0135959 | Organic acids and derivatives | NEG |
| 646 | Xanthosine | 0.662343230769231 | 312.0735 | 282.9992014 | Nucleosides, nucleotides, and analogues | NEG |
| 647 | 5-hydroxy-10,10-dimethyl-6-methylidenebicyclo[7.2.0]undecan-2-one | 0.655807846153846 | 28.609 | 220.8861288 | Organic oxygen compounds | NEG |
| 648 | 1-[(2R,4S,5R)-4-hydroxy-5-(hydroxymethyl)oxolan-2-yl]-5-methylpyrimidine-2,4-dione | 0.654746769230769 | 343.661 | 240.1595499 | Nucleosides, nucleotides, and analogues | NEG |
| 649 | Uracil | 0.643863384615385 | 233.074 | 111.0189782 | Organoheterocyclic compounds | NEG |
| 650 | 3-hydroxybutanoic acid | 0.629403076923077 | 642.3865 | 103.0390648 | Organic acids and derivatives | NEG |
| 651 | 2-(1H-indol-3-yl)acetonitrile | 0.619723923076923 | 41.9133 | 155.0007106 | Organoheterocyclic compounds | NEG |
| 652 | (2E)-Decenoyl-ACP | 0.619497846153846 | 313.8195 | 129.0375468 | Organic acids and derivatives | NEG |
| 653 | (1R,2S,3S,4R,5R)-6,8-dioxabicyclo[3.2.1]octane-2,3,4-triol | 0.619475307692308 | 334.572 | 160.0797818 | Organoheterocyclic compounds | NEG |
| 654 | NAD | 0.601203769230769 | 447.7985 | 662.1021338 | Nucleosides, nucleotides, and analogues | NEG |
| 655 | (2S)-2-amino-5-(diaminomethylideneamino)pentanoic acid | 0.595975 | 532.66 | 173.1034889 | Organic acids and derivatives | NEG |
| 656 | Gluconic acid | 0.592104 | 388.499 | 195.0500976 | Organic oxygen compounds | NEG |
| 657 | LysoPA(18:1(9Z)/0:0) | 0.582413769230769 | 187.1265 | 434.9853618 | Lipids and lipid-like molecules | NEG |
| 658 | Pterin | 0.579180615384615 | 218.566 | 162.0220269 | Organoheterocyclic compounds | NEG |
| 659 | (1R,2S,3R)-2-Acetyl-4(5)-(1,2,3,4-tetrahydroxybutyl)imidazole | 0.572897769230769 | 328.1205 | 229.0820716 | Organic oxygen compounds | NEG |
| 660 | 1-Deoxy-D-xylulose 5-phosphate | 0.567632461538462 | 170.242 | 212.8384424 | Organic oxygen compounds | NEG |
| 661 | 2-(3,4-dihydroxyphenyl)-3,5,7-trihydroxychromen-4-one | 0.567255384615385 | 97.86985 | 336.90915 | Phenylpropanoids and polyketides | NEG |
| 662 | (2R,4R)-pentane-1,2,3,4,5-pentol | 0.559540307692308 | 252.868 | 151.0601211 | Organic oxygen compounds | NEG |
| 663 | Maltotetraose | 0.556027769230769 | 507.919 | 665.2138174 | Organic oxygen compounds | NEG |
| 664 | Lipoxin B4 | 0.555926846153846 | 112.198 | 352.1732415 | Lipids and lipid-like molecules | NEG |
| 665 | (Z)-tetradec-9-enoic acid | 0.549247615384615 | 49.8165 | 225.9927883 | Lipids and lipid-like molecules | NEG |
| 666 | Myricetin | 0.540816 | 108.085 | 316.1988755 | Phenylpropanoids and polyketides | NEG |
| 667 | Mesaconic acid | 0.538451615384615 | 408.1925 | 129.0376416 | Lipids and lipid-like molecules | NEG |
| 668 | Aminoadipic acid | 0.535997 | 217.414 | 160.0064493 | Organic acids and derivatives | NEG |
| 669 | Iminodiacetic acid | 0.529576076923077 | 292.2285 | 131.0896945 | Organic acids and derivatives | NEG |
| 670 | (2R,3R)-2-(3,4-dihydroxyphenyl)-3,4-dihydro-2H-chromene-3,5,7-triol | 0.524446769230769 | 101.683 | 288.0908315 | Phenylpropanoids and polyketides | NEG |
| 671 | 2-oxohexanedioic acid | 0.522352461538462 | 347.197 | 158.9878482 | Organic acids and derivatives | NEG |
| 672 | Deoxyribose 5-phosphate | 0.513788230769231 | 71.1742 | 212.0745316 | Organic oxygen compounds | NEG |
| 673 | 6-(trifluoromethoxy)-1,3-benzothiazol-2-amine | 0.510872538461538 | 244.6385 | 232.044205 | Organoheterocyclic compounds | NEG |
| 674 | [(2R,3S,5R)-3-hydroxy-5-(6-oxo-1H-purin-9-yl)oxolan-2-yl]methyl dihydrogen phosphate | 0.494252692307692 | 373.299 | 331.0207133 | Nucleosides, nucleotides, and analogues | NEG |
| 675 | Sedoheptulose | 0.463974384615385 | 171.417 | 208.8443673 | Organic oxygen compounds | NEG |
| 676 | Valdecoxib | 0.438938846153846 | 107.006 | 312.071671 | Benzenoids | NEG |
| 677 | beta-D-Glucosamine | 0.435021923076923 | 451.975 | 214.0112031 | Organooxygen compounds | NEG |
| 678 | (2S)-2-[(2-aminoacetyl)amino]-3-(4-hydroxyphenyl)propanoic acid | 0.389363692307692 | 280.878 | 236.1043544 | Organic acids and derivatives | NEG |
| 679 | N-[4-cyano-3-(trifluoromethyl)phenyl]-3-(4-fluorophenyl)sulfonyl-2-hydroxy-2-methylpropanamide | 0.376531 | 231.386 | 429.0548739 | Benzenoids | NEG |
| 680 | 2-amino-3-methylpentanoic acid | 0.999946846153846 | 292.2195 | 130.0862684 | Organic acids and derivatives | NEG |
| 681 | 3,7-dihydropurin-6-one | 0.999895153846154 | 231.7465 | 137.0452217 | Organoheterocyclic compounds | POS |
| 682 | Inosine | 0.999834615384615 | 260.799 | 268.1503266 | Nucleosides, nucleotides, and analogues | POS |
| 683 | 2-amino-9-[(2R,4S,5R)-4-hydroxy-5-(hydroxymethyl)oxolan-2-yl]-1H-purin-6-one | 0.999724615384615 | 164.875 | 268.0606412 | Nucleosides, nucleotides, and analogues | POS |
| 684 | 5-oxopyrrolidine-2-carboxylic acid | 0.999627307692308 | 313.8195 | 128.0341747 | Organic acids and derivatives | NEG |
| 685 | 7H-purin-6-amine | 0.999466538461538 | 397.947 | 136.0612694 | Organoheterocyclic compounds | POS |
| 686 | 2-amino-3-methyl-4H-imidazol-5-one | 0.999171153846154 | 91.2486 | 113.5330601 | Organic acids and derivatives | POS |
| 687 | 1-[(2R,3R,4S,5R)-3,4-dihydroxy-5-(hydroxymethyl)oxolan-2-yl]pyrimidine-2,4-dione | 0.998104384615385 | 164.363 | 243.0616107 | Nucleosides, nucleotides, and analogues | NEG |
| 688 | Adenine | 0.998096923076923 | 422.611 | 136.0612702 | Organoheterocyclic compounds | POS |
| 689 | gamma-Glutamylleucine | 0.998094692307692 | 361.327 | 262.1259949 | Organic acids and derivatives | POS |
| 690 | 2-aminoethanesulfonic acid | 0.997931076923077 | 311.896 | 126.0214838 | Organic acids and derivatives | POS |
| 691 | (2S)-2-aminopentanedioic acid | 0.991784307692308 | 381.919 | 147.9974814 | Organic acids and derivatives | POS |
| 692 | Argininosuccinic acid | 0.990878615384615 | 455.245 | 291.0803196 | Organic acids and derivatives | POS |
| 693 | Uridine | 0.990189461538462 | 165.7065 | 245.0754791 | Nucleosides, nucleotides, and analogues | POS |
| 694 | L-Aspartic acid | 0.988818230769231 | 414.728 | 132.0292089 | Organic acids and derivatives | NEG |
| 695 | (2S)-2-aminobutanedioic acid | 0.985605923076923 | 390.8075 | 134.0636542 | Organic acids and derivatives | POS |
| 696 | Carnosine | 0.982457846153846 | 438.415 | 226.1790343 | Organic acids and derivatives | POS |
| 697 | (2R,3R,4S,5S)-2-(6-aminopurin-9-yl)-5-(methylsulfanylmethyl)oxolane-3,4-diol | 0.977837307692307 | 53.0664 | 297.142921 | Nucleosides, nucleotides, and analogues | POS |
| 698 | Deoxyadenosine | 0.977757 | 97.5176 | 251.173953 | Nucleosides, nucleotides, and analogues | POS |
| 699 | (2S)-2-amino-3-(4-hydroxyphenyl)propanoic acid | 0.976374769230769 | 340.815 | 180.0657277 | Organic acids and derivatives | NEG |
| 700 | LysoPE(18:1(9Z)/0:0) | 0.970560307692308 | 220.234 | 480.3068722 | Lipids and lipid-like molecules | POS |
| 701 | 4-aminobutanoic acid | 0.966699923076923 | 91.2039 | 103.5478772 | Organic acids and derivatives | POS |
| 702 | Risedronate | 0.963243846153846 | 381.107 | 284.007745 | Organic acids and derivatives | POS |
| 703 | Protoporphyrin IX | 0.947534769230769 | 165.025 | 563.0673717 | Organoheterocyclic compounds | POS |
| 704 | [[(2R,3S,4R,5R)-5-(4-amino-2-oxopyrimidin-1-yl)-3,4-dihydroxyoxolan-2-yl]methoxy-hydroxyphosphoryl] 2-(trimethylazaniumyl)ethyl phosphate | 0.944628384615385 | 459.462 | 486.2671683 | Nucleosides, nucleotides, and analogues | NEG |
| 705 | Uridine 5'-monophosphate | 0.929953076923077 | 454.1515 | 325.0415328 | Nucleosides, nucleotides, and analogues | POS |
| 706 | (2S)-2-amino-4-[[(2S,3S,4R,5R)-5-(6-aminopurin-9-yl)-3,4-dihydroxyoxolan-2-yl]methylsulfanyl]butanoic acid | 0.912364230769231 | 382.689 | 384.9753307 | Nucleosides, nucleotides, and analogues | POS |
| 707 | (2S)-2,5-diamino-5-oxopentanoic acid | 0.907508307692308 | 36.70515 | 146.9798022 | Organic acids and derivatives | POS |
| 708 | (2S,3R)-2-amino-3-hydroxybutanoic acid | 0.843061076923077 | 367.013 | 118.0499064 | Organic acids and derivatives | NEG |
| 709 | Riluzole | 0.778367153846154 | 205.731 | 232.9501847 | Organoheterocyclic compounds | NEG |
| 710 | 3-Nitrotyrosine | 0.727739538461538 | 141.378 | 225.0255867 | Organic acids and derivatives | NEG |
| 711 | (2S,4R)-4-hydroxy-1-[(2S)-pyrrolidin-1-ium-2-carbonyl]pyrrolidine-2-carboxylate | 0.696236076923077 | 427.382 | 229.0097713 | Organic acids and derivatives | POS |
| 712 | N-[1-[(2R,3R,4S,5R)-3,4-dihydroxy-5-(hydroxymethyl)oxolan-2-yl]-2-oxopyrimidin-4-yl]acetamide | 0.615884230769231 | 189.811 | 283.2632811 | Nucleosides, nucleotides, and analogues | NEG |

MS2 Score: mass-spectrometry 2 Score; rt: retention time; mz: mass-to-charge ratio;

**Table S2** 77 genes mRNA expression of Glycerophospholipid metabolism among all ESCC TNM stages and adjacent normal control tissues.

| **Glycerophospholipid metabolic pathway genes** | **Significantly differential mRNA expression** |
| --- | --- |
| ACHE | No |
| ADPRM | No |
| AGPAT1 | No |
| AGPAT2 | No |
| AGPAT3 | No |
| AGPAT4 | No |
| CDIPT | Yes |
| CDS1 | No |
| CDS2 | No |
| CHAT | No |
| CHKA | Yes |
| CHKB | No |
| CHPT1 | No |
| CRLS1 | No |
| DGKA | No |
| DGKB | No |
| DGKD | No |
| DGKE | No |
| DGKG | No |
| DGKH | Yes |
| DGKI | No |
| DGKQ | No |
| DGKZ | Yes |
| ETNK1 | No |
| ETNK2 | No |
| GNPAT | No |
| GPAM | No |
| GPAT2 | No |
| GPAT3 | No |
| GPAT4 | No |
| GPD1 | No |
| GPD1L | No |
| GPD2 | No |
| JMJD7-PLA2G4B | No |
| LCAT | Yes |
| LCLAT1 | Yes |
| LPCAT1 | Yes |
| LPCAT2 | Yes |
| LPCAT3 | No |
| LPCAT4 | No |
| LPGAT1 | Yes |
| LYPLA1 | No |
| LYPLA2 | No |
| MBOAT1 | No |
| MBOAT2 | No |
| MBOAT7 | Yes |
| PCYT1A | Yes |
| PCYT1B | No |
| PCYT2 | No |
| PEMT | Yes |
| PGS1 | Yes |
| PHOSPHO1 | No |
| PISD | Yes |
| PLA2G10 | No |
| PLA2G12A | No |
| PLA2G12B | No |
| PLA2G15 | Yes |
| PLA2G1B | No |
| PLA2G2A | No |
| PLA2G2C | No |
| PLA2G2D | No |
| PLA2G2E | No |
| PLA2G2F | No |
| PLA2G3 | No |
| PLA2G4A | No |
| PLA2G4B | No |
| PLA2G4E | No |
| PLA2G5 | No |
| PLA2G6 | No |
| PLD1 | No |
| PLD2 | No |
| PLPP1 | No |
| PLPP2 | No |
| PLPP3 | No |
| PTDSS1 | Yes |
| PTDSS2 | No |
| TAZ | No |
| Total genes (77) | Total genes that differed significantly in content (16) |
